# Dietary Bioactives and Physical Activity in the Regulation of Hippocampal Neurogenesis and Cognitive Decline

**DOI:** 10.1002/fsn3.71613

**Published:** 2026-03-09

**Authors:** Zhenyi Zhao, Sima‐sadat Sabihi

**Affiliations:** ^1^ School of Competitive Sports Beijing Sport University Beijing China; ^2^ Food Security Research Center Isfahan University of Medical Sciences Isfahan Iran

**Keywords:** cognitive decline, hippocampus, natural products, neurogenesis, physical exercise

## Abstract

Aging and neurodegenerative disorders are distinguished by dysfunction within the hippocampus, resulting in compromised spatial memory and cognitive deterioration. The modulation of neurogenesis and neuroinflammation has surfaced as a promising strategy to restore hippocampal functionality and enhance cognitive capabilities. Natural substances, encompassing polyphenols, flavonoids, and various bioactive compounds, display neuroprotective, antioxidant, and anti‐inflammatory effects, whereas physical exercise promotes neurogenesis, synaptic plasticity, and anti‐inflammatory pathways. Recent findings indicate that the integration of dietary modifications alongside regular physical activity yields synergistic advantages for preserving cerebral health and alleviating cognitive decline. This review consolidates existing knowledge regarding the molecular mechanisms by which natural substances and physical activity impact hippocampal neurogenesis and neuroinflammation. It emphasizes critical pathways, including the regulation of neurotrophic factors, reduction of oxidative stress, and modulation of inflammatory cytokines, which collectively underpin cognitive functionality. Through the amalgamation of nutritional and lifestyle interventions, this methodology presents prospective preventive and therapeutic advantages for geriatric demographics and individuals afflicted with neurodegenerative conditions. Comprehending the intricate relationship between dietary habits and physical activity in relation to cerebral health may facilitate the formulation of accessible, non‐pharmacological strategies aimed at augmenting hippocampal functionality, safeguarding memory retention, and enhancing overall cognitive efficacy.

## Graphical abstract

Exercise and dietary polyphenols enhance hippocampal plasticity and cognitive function via complementary molecular mechanisms. Central pathways include BDNF signaling, inflammation control, and mitochondrial regulation. Although synergy is mainly supported by preclinical data, lifestyle‐based strategies show promise for brain health maintenance.

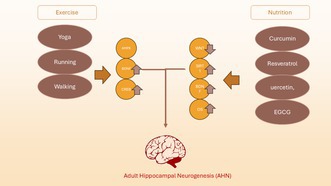

**Abbreviations**
AD: Alzheimer's disease
AHN: adult hippocampal neurogenesis
AMPK: AMP‐activated protein kinase
BDNF: brain‐derived neurotrophic factor
BrdU: bromodeoxyuridine
CI: cognitive impairment
DCX: doublecortin
EGCG: epigallocatechin gallate
HIIT: high‐intensity interval training
IL‐6: interleukin‐6
MAPK: mitogen‐activated protein kinase
MICT: moderate‐intensity continuous training
mTOR: mammalian target of rapamycin
NF‐κB: nuclear factor kappa B
Nrf2: nuclear factor erythroid 2–related factor 2
NSCs: neural stem cells
PI3K: phosphatidylinositol 3‐kinase
SGZ: subgranular zone
SIRT1: sirtuin 1
SVZ: subventricular zone
TNF‐α: tumor necrosis factor‐alpha
VEGF: vascular endothelial growth factor

## 1 Introduction

The global population of older adults is increasing rapidly, a trend projected to accelerate in the coming decades (Bhattacharjee et al. 2023; Eshkoor et al. 2015; Pais et al. 2020). By 2035, the number of individuals aged 60 and above is expected to rise by approximately 56%, whereas those over 80 are predicted to triple by 2050 (Bhattacharjee et al. 2023; Culig et al. 2022; Pérez Palmer et al. 2022; Selles et al. 2018). Cognitive impairment (CI), manifesting as memory deficits, reduced learning capacity, and attention difficulties, is now recognized as a significant public health concern, affecting over 55 million people worldwide, with approximately 10 million new cases each year (Johansson et al. 2015; Ren et al. 2018). As populations age, CI has become a major cause of dependency and reduced quality of life in older adults (Johansson et al. 2015). Multiple factors contribute to the development of CI, including genetic predisposition, environmental exposures, and lifestyle‐related risks (Sun et al. 2010). Key contributing mechanisms include neurodegenerative changes, cerebrovascular pathology, and stroke (Badji et al. 2023; Huang et al. 2022). CI imposes not only personal and medical burdens but also significant socioeconomic costs, as affected individuals often lose independence and require ongoing care (Frota et al. 2016).

Contrary to long‐standing assumptions, the adult brain retains the ability to generate new neurons. This process, termed adult neurogenesis, primarily occurs in two regions: the subventricular zone (SVZ) and the subgranular zone (SGZ) of the dentate gyrus in the hippocampus (Oomen et al. 2014). Neurons produced in the SGZ integrate into hippocampal circuits, where they contribute to learning, memory, and emotional regulation (Toda et al. 2019). The hippocampus plays a critical role in memory formation and spatial navigation, and newly generated neurons are essential for sustaining its plasticity (Salta et al. 2023; Slotnick 2022). Adult hippocampal neurogenesis (AHN) is characterized by the differentiation of neural stem cells (NSCs) into functional neurons, which exhibit enhanced synaptic plasticity and excitability during integration (Tartt et al. 2018; Vivar et al. 2013). AHN is highly responsive to external stimuli such as physical activity, environmental enrichment, and cognition‐enhancing pharmacological agents (Boldrini et al. 2018). Genetic and pharmacological studies have shown that enhancing neurogenesis improves cognitive performance in both healthy and diseased states, positioning AHN as a potential therapeutic target in age‐related cognitive decline and neurodegenerative disorders (Gao et al. 2023).

Physical exercise is a well‐established modulator of brain plasticity. It can be broadly categorized into general physical activity and structured physical exercise (Caspersen et al. 1985; Ribarič 2022). Although physical activity refers to any movement requiring energy expenditure, physical exercise involves systematic and goal‐oriented training that improves physical fitness (Sanaeifar et al. 2024). Various forms of physical exercise have demonstrated significant benefits for brain health, particularly aerobic exercise, which has shown the most robust evidence in slowing the progression of neurodegenerative conditions like Parkinson's disease (Purtle et al. 2020). Exercise promotes neuroplasticity by increasing neurotrophic factors such as brain‐derived neurotrophic factor (BDNF), enhancing cognitive performance, and supporting structural brain changes. Activities such as brisk walking, dancing, yoga, and high‐intensity interval training (HIIT) have all been linked to improved cognition and mood stability (Liu et al. 2020; Nokia et al. 2016; Sanaeifar et al. 2024; Van Praag et al. 1999). Consistent aerobic training appears especially effective in stimulating AHN, particularly in individuals genetically predisposed to benefit from physical activity (Liu et al. 2020; Nokia et al. 2016; Sanaeifar et al. 2024; Van Praag et al. 1999). In parallel, nutritional strategies, particularly those involving polyphenols, are gaining attention for their neuroprotective effects. Polyphenols are bioactive compounds widely found in plant‐based foods such as fruits, vegetables, tea, and wine (Cicero et al. 2018; Potì et al. 2019; Wahl et al. 2016). Observational and clinical studies suggest that polyphenol‐rich diets may help preserve cognitive function and reduce the risk of neurodegeneration in aging individuals (Pervin et al. 2018; Potì et al. 2019). The mechanisms underlying polyphenols' benefits include antioxidant activity, anti‐inflammatory effects, and modulation of cellular pathways involved in brain aging (Abbott et al. 2010; Chung et al. 2010; Flanagan et al. 2018; Khurana et al. 2013; Rahman et al. 2006; Ramis, Sarubbo, Moranta, et al. 2020; Ramis, Sarubbo, Tejada, et al. 2020). Regular intake of polyphenol‐rich foods has been associated with improvements in memory, attention, and overall cognitive performance (Angelino et al. 2019).

Emerging evidence indicates that the combination of polyphenol supplementation and exercise may yield synergistic benefits. This dual intervention has been shown to reduce oxidative stress markers more effectively than either intervention alone. In preclinical models, co‐treatment with polyphenols (e.g., curcumin) and exercise significantly attenuated neurotoxic damage and enhanced antioxidant defense. These findings highlight a promising strategy for enhancing brain resilience and preventing age‐related cognitive decline (Amirazodi et al. 2022; Hosseinzadeh et al. 2013).

This review explores the intersection of structured exercise, polyphenol intake, and AHN in the context of aging and neurodegenerative disease. We aim to synthesize current evidence on how these interventions influence molecular pathways involved in cognition, and to identify non‐pharmacological strategies that support brain health during aging. Although substantial mechanistic insight has been obtained from preclinical models, the extent to which these findings translate to humans remains an active area of investigation. This review therefore distinguishes between evidence derived from animal studies and emerging clinical data when discussing the cognitive and neurogenic effects of exercise and dietary polyphenols.

## 2 Materials and Methods (Search Strategy and Selection Criteria)

This narrative review was conducted to summarize and integrate current evidence on the role of diet‐derived natural products and physical activity in modulating hippocampal neurogenesis, neuroinflammation, and cognitive function during aging and neurodegenerative diseases. The literature search was performed using the electronic databases PubMed/MEDLINE, Scopus, and Web of Science to identify relevant peer‐reviewed publications in the fields of food science, nutrition, neuroscience, and aging.

Search terms were selected to capture studies examining bioactive food compounds, lifestyle interventions, and hippocampal plasticity. Keywords included combinations of “hippocampal neurogenesis,” “adult neurogenesis,” “physical exercise,” “physical activity,” “natural products,” “polyphenols,” “flavonoids,” “dietary bioactives,” “functional foods,” “neuroinflammation,” “aging,” “cognitive decline,” and “neurodegenerative diseases.” Boolean operators (“AND,” “OR”) were used to refine searches. Only articles published in English were considered, with no strict start date, and the search included the most recent literature available at the time of manuscript preparation.

Studies were included if they met the following criteria: (i) original experimental studies, clinical trials, or relevant review articles published in peer‐reviewed journals; (ii) investigations evaluating the effects of food‐derived natural products, dietary patterns, or physical exercise on hippocampal neurogenesis, neuroinflammatory pathways, or cognitive outcomes; and (iii) studies involving aging models, older adult populations, or neurodegenerative disease contexts. Exclusion criteria included conference abstracts, editorials, case reports, non‐peer‐reviewed sources, and studies not directly addressing hippocampal mechanisms or cognition.

Article selection was based on screening of titles and abstracts, followed by full‐text assessment for relevance to the objectives of the review. Particular emphasis was placed on studies elucidating nutritional and molecular mechanisms, including antioxidant activity, modulation of inflammatory cytokines, regulation of neurotrophic factors such as BDNF, and interactions between dietary bioactives and exercise‐induced signaling pathways. As this is a narrative review, a formal systematic review protocol or meta‐analysis was not applied; instead, the selected literature was qualitatively synthesized to provide a mechanistic and nutrition‐oriented perspective on how combined dietary and physical activity interventions may support hippocampal function and cognitive health during aging.

## 3 Definition and Process of Neurogenesis

Neurogenesis refers to the biological process through which new neurons are generated from NSCs (Ribeiro and Xapelli 2021). Initially believed to occur only during embryonic and early postnatal brain development, it is now well established that neurogenesis continues into adulthood, albeit in a more restricted and tightly regulated manner (Seki et al. 2019). Pioneering studies, particularly those by Eriksson et al. (1998), and later confirmed by Spalding et al. (2013), demonstrated ongoing neuronal generation in the adult human brain. These findings shifted the long‐standing paradigm that the adult brain lacked regenerative potential (Eriksson et al. 1998; Spalding et al. 2013). Adult neurogenesis primarily occurs in two specific neurogenic niches: SGZ of the dentate gyrus in the hippocampus and SVZ along the lateral ventricles (Hagg 2009; Wu and Zhang 2023). These regions contain quiescent NSCs that can be activated under physiological or pathological conditions. Additional brain areas, such as the striatum and amygdala, have shown signs of neurogenic activity in some models, although evidence in these regions remains limited and under investigation (Villalba et al. 2021).

The hippocampus, which is critical for memory consolidation, spatial navigation, learning, and emotional regulation, is one of the best‐characterized regions for adult neurogenesis. In the SGZ, the process begins with radial‐glia‐like Type 1 NSCs, which have self‐renewing and multipotent capabilities. Upon activation, these cells divide asymmetrically to produce intermediate progenitor cells (Type 2a and 2b), each with distinct molecular and morphological profiles. These progenitors subsequently differentiate into Type 3 neuroblasts—postmitotic, migratory precursors committed to the neuronal lineage (Anand and Dhikav 2012; Flor‐García et al. 2020). The regulation of adult neurogenesis is multifactorial. Intrinsic mechanisms involve transcription factors, intracellular signaling cascades, and epigenetic regulators that guide cell fate decisions. Extrinsic influences originate from the local microenvironment and include neurotransmitters (e.g., serotonin, dopamine), growth factors (e.g., BDNF), hormones (e.g., cortisol, estrogen), immune mediators (e.g., cytokines), and metabolic signals (Wu and Zhang 2023). Modifiable lifestyle factors such as physical activity, environmental enrichment, diet, stress levels, and sleep quality can profoundly influence the rate and quality of neurogenesis (Barone 2024; Du Preez et al. 2024). Although the extent of neurogenesis in the adult human brain, especially in the elderly, remains a topic of ongoing debate, several advanced techniques such as carbon‐14 birth dating, bromodeoxyuridine (BrdU) labeling, immunohistochemistry, and single‐cell RNA sequencing support its persistence into later life (Lucassen et al. 2020). However, the rate of neurogenesis appears to decline with aging and may be further reduced by neurodegenerative diseases, chronic stress, and systemic inflammation (Culig et al. 2022). Importantly, impaired or dysregulated neurogenesis has been implicated in various neurological and psychiatric disorders, including Alzheimer's disease (AD), major depressive disorder, schizophrenia, and epilepsy. Reduced neurogenesis in these conditions may contribute to cognitive deficits, emotional disturbances, and heightened vulnerability to stress. Conversely, interventions that enhance neurogenesis, whether pharmacological, genetic, or behavioral—are being actively explored for their therapeutic potential in brain repair and regeneration (Hagihara et al. 2019). Thus, adult neurogenesis is a complex, multi‐step process that reflects the brain's capacity for plasticity and functional adaptation. By generating neurons that can integrate into preexisting circuits, neurogenesis supports learning, memory, emotional regulation, and possibly recovery from injury. A deeper understanding of the molecular and environmental factors regulating this process could lead to innovative therapies aimed at preventing or reversing cognitive decline and treating neurodegenerative and psychiatric conditions.

## 4 Physical Exercise as a Natural Approach to Boost Neurogenesis

The discovery of adult neurogenesis, particularly in the hippocampus, has significantly reshaped our understanding of brain plasticity. Once believed to occur only during early development, it is now recognized as a dynamic process contributing to cognitive and emotional health throughout life (Cole et al. 2019). Adult NSCs not only support tissue maintenance but also enhance brain adaptability, playing a central role in learning, memory, and mood regulation (Bond et al. 2015). The dentate gyrus of the hippocampus is one of the most extensively studied regions for adult neurogenesis. Here, newly generated neurons integrate into existing neural circuits and contribute to processes such as memory encoding and emotional control. Diminished neurogenesis in this region has been linked to mood disorders, including depression. As shown in animal models, decreased AHN is associated with depressive‐like behavior, whereas antidepressant treatments can stimulate neuronal proliferation—suggesting a potential role for neurogenesis in recovery from depression (Becker and Wojtowicz 2007). Maintaining neurogenesis into old age is increasingly recognized as a critical factor in preserving cognitive function. Boldrini et al. (2018) reported that healthy older individuals continue to generate new hippocampal neurons, even as other neurobiological systems decline with age. This ongoing capacity may help explain why some elderly individuals retain high cognitive performance despite age‐related neurovascular changes (Boldrini et al. 2018). Neurogenesis also appears to be sensitive to acute insults. For example, cognitive deficits following surgical procedures have been linked to suppressed AHN. Fan et al. (2016) demonstrated that an enriched environment, known to elevate BDNF, can mitigate postoperative learning and memory impairments, likely by promoting neurogenesis (Fan et al. 2016).

At the molecular level, several key regulatory pathways influence the efficiency of neurogenesis with age. Hu et al. (2022) identified the RNA‐binding protein LIN28A as a mediator of Wnt signaling, a pathway crucial for neuronal development. Age‐related declines in LIN28A expression were shown to impair neuronal integration and reduce pattern separation, an essential cognitive function for distinguishing similar experiences. Enhancing LIN28A or related signaling components may offer therapeutic benefits in age‐related memory decline (Hu et al. 2022). Autophagy, a cellular process that clears damaged organelles and maintains homeostasis, also plays an important role in supporting neurogenesis. Yang et al. (2022) found that stimulating autophagy in middle‐aged mice revitalized neural precursor cells and improved cognitive performance. These findings suggest that promoting autophagic activity may counteract age‐related reductions in neurogenesis (Yang et al. 2022). The relationship between neurogenesis and neurodegenerative diseases is particularly prominent in AD. As shown by Zhou et al. (2023), AD progression is associated with reduced hippocampal neurogenesis and diminished stem cell proliferation, contributing to the cognitive and emotional symptoms characteristic of the disease. These findings underscore the potential of neurogenesis as both a biomarker and a therapeutic target in neurodegenerative disorders (Zhou et al. 2023).

Beyond cognition, neurogenesis is also involved in emotional regulation. Alonso et al. (2024) reported that new neurons integrate into circuits that modulate mood and affective responses, highlighting neurogenesis as a cellular mechanism through which internal states and external stimuli influence behavior. This connection suggests that enhancing neurogenesis could serve as a novel approach in treating emotional and stress‐related disorders (Alonso et al. 2024). Lifestyle factors, especially physical activity and diet, are strong modulators of neurogenesis and play a key role in brain aging. Du Preez et al. (2024) proposed a neurogenesis‐centered model of brain health, suggesting that the influence of nutrition, exercise, and other modifiable behaviors on cognitive aging may be largely mediated through their effects on neurogenesis. This model supports the idea that stimulating neuronal renewal could help delay or reduce the impact of cognitive decline, depression, and dementia (Du Preez et al. 2024). Despite growing interest in neurogenesis as a therapeutic target, standardizing methods for measuring this process remains a challenge. Zhao and van Praag (2020) emphasized the importance of consistent and accurate quantification techniques to facilitate translation of basic research into clinical applications (Zhao and van Praag 2020). Therefore, adult hippocampal neurogenesis plays a vital role in memory, learning, emotional resilience, and neuroprotection. Although aging and disease can impair this process, various interventions, including environmental enrichment, exercise, dietary bioactives, autophagy enhancement, and molecular modulation, offer promising strategies to preserve or restore cognitive health across the lifespan.

The production of new neurons in the adult brain, particularly within the hippocampus, has dramatically shifted our understanding of brain function and plasticity. Previously believed to be a developmental process limited to early life, adult neurogenesis is now recognized as a key contributor to maintaining cognitive and emotional well‐being (Cole et al. 2019). As highlighted by Bond et al. (2015), adult NSCs not only sustain tissue renewal but also enhance brain adaptability, showing promise in supporting lifelong mental health (Bond et al. 2015). One of the most studied neurogenic regions is the dentate gyrus of the hippocampus, where newly formed neurons play a critical role in learning, memory formation, and emotional regulation. Research by Becker and Wojtowicz (2007) emphasized the link between diminished neurogenesis and mood disorders such as depression. In animal studies, decreased hippocampal neurogenesis is associated with depressive‐like behaviors, whereas antidepressant treatments often stimulate the growth of new neurons, suggesting that neurogenesis might be central to recovery (Becker and Wojtowicz 2007).

Maintaining neurogenesis during aging is another key factor in preserving cognitive integrity. Contrary to earlier assumptions that neuron production stops in adulthood, Boldrini et al. (2018) found that healthy older individuals retain the ability to generate new neurons in the hippocampus (Boldrini et al. 2018). Although neurovascular factors and cellular plasticity decline with age, the persistence of neural progenitors may help explain the resilience seen in some elderly people who remain cognitively intact. CI, including that caused by surgical procedures, also appears to be linked with disruptions in hippocampal neurogenesis. Fan et al. (2016) demonstrated that an enriched environment, known to increase BDNF, can mitigate learning and memory deficits after surgery, likely by supporting ongoing neurogenesis (Fan et al. 2016). Furthermore, molecular pathways regulating neurogenesis become increasingly important with age. Hu et al. (2022) identified LIN28A, an RNA‐binding protein, as a crucial mediator in the Wnt signaling pathway. Its age‐related decline was shown to impair the generation and integration of new neurons, particularly affecting pattern separation, a cognitive function essential for distinguishing between similar memories. These findings imply that supporting neurogenesis at a molecular level may improve age‐related cognitive deficits (Hu et al. 2022). Another mechanism shown to support neural regeneration is autophagy, the cellular process that clears damaged components and maintains homeostasis. According to Yang et al. (2022), stimulating autophagy in middle‐aged mice revitalized neural precursor cells and restored cognitive performance, pointing to autophagy as a potential therapeutic target to counteract age‐related neurogenesis decline (Yang et al. 2022).

Thus, neurogenesis in the adult hippocampus is a vital process that underlies memory, learning, emotional balance, and resilience against neurodegeneration. Although aging and disease can impair this process, various strategies, including environmental enrichment, dietary interventions, autophagy activation, and molecular modulation, offer promising avenues for preserving or restoring cognitive health (Table 1).

**TABLE 1:** Summary of preclinical studies on exercise‐induced neurogenesis and cognitive function.

Condition/disease	Study type	Exercise/treatment	Mechanisms involved	Key outcomes	References
Natural aging‐related cognitive decline	Pre‐clinical	Aerobic, resistance, or combined exercise (12 weeks)	Upregulation of Notch signaling and autophagy (LC3, Beclin1, p62); increased neurogenesis (DCX, Ki67, GFAP)	Improved memory and enhanced neurogenic and autophagic marker expression	Chen et al. (2025)
Brain aging	Pre‐clinical	Endurance exercise	Increased autophagy‐related proteins; activation of NRG1‐ERK‐RSK‐CREB signaling	Enhanced neurogenesis and synaptic plasticity	Jang (2020)
Maternal obesity‐induced memory decline	Pre‐clinical	Treadmill exercise during pregnancy	Increased BDNF and TrkB expression; enhanced hippocampal neurogenesis	Improved offspring memory; elevated BrdU and DCX levels	Ji et al. (2020)
Alzheimer‐like impairment in diabetes	Pre‐clinical	Treadmill exercise (12 weeks)	Activation of Wnt signaling; inhibition of GSK‐3β	Improved spatial memory and learning; increased BrdU and DCX	Kim et al. (2016)
Depression from chronic stress	Pre‐clinical	Exercise ± VEGF receptor inhibitor	VEGF‐Flk‐1 signaling; increased neurogenesis and vascular density	Behavioral improvement; neurogenesis reversed by VEGF inhibition	Kiuchi et al. (2012)
General cognitive enhancement	Pre‐clinical	Resistance wheel training (14 days)	Elevated BDNF, CREB; mTOR‐p70S6K pathway activation in muscle	Improved spatial memory; mTOR signaling correlated with hippocampal BDNF	Suijo et al. (2013)
Cerebral palsy	Pre‐clinical	Treadmill running (6 weeks)	Activation of PI3K‐Akt‐Wnt; suppression of GSK‐3β and β‐catenin	Increased hippocampal cell proliferation; reduced apoptosis; improved memory	Cho et al. (2020)
PTSD	Pre‐clinical	Treadmill running	Akt signaling and hippocampal neurogenesis	Enhanced cognition and neurogenesis; blocked by Akt inhibition	Sun et al. (2020)
Cognitive modulation in healthy brains	Pre‐clinical	Mild vs. intense treadmill exercise	Corticosterone modulation; gene regulation (lipid metabolism, inflammation)	Moderate‐intensity improved memory and neurogenesis; intense impaired it	Inoue et al. (2015)
Cognitive resilience post‐adrenalectomy	Pre‐clinical	Treadmill running + MR antagonist	Glucocorticoid signaling; MR downregulation	Exercise‐induced neurogenesis partly dependent on MR modulation	Chang et al. (2008)
Depression	Pre‐clinical	Ginsenoside Rg1 administration	BDNF pathway activation; reduced corticosterone	Antidepressant‐like effects via neurogenesis	Jiang et al. (2012)
Alzheimer's disease	Pre‐clinical	Treadmill running	MAPK signaling modulation; reduced inflammation	Improved memory; reduced Aβ‐induced deficits and hippocampal inflammation	Sun et al. (2018)
Huntington's disease	Pre‐clinical	Voluntary running + enrichment	Akt signaling deficiency; GH/IGF‐1 axis	Impaired neurogenesis despite exercise; reduced Akt phosphorylation	Ransome and Hannan (2013)
Post‐stroke depression	Pre‐clinical	CUMS in ischemic rats	Suppressed Notch1 (NICD, Hes1, Hes5) signaling	Decreased neurogenesis and altered cell fate after injury and stress	Guo et al. (2009)
Cognitive performance	Pre‐clinical	High‐intensity interval training (HIIT)	BDNF upregulation	Improved spatial memory and neurogenesis	Okamoto et al. (2021)
Major depression	Pre‐clinical	NPY1R agonist + ketamine	TrkB and NPY1R complex formation; BDNF increase	Synergistic antidepressant effects; enhanced hippocampal neurogenesis	Arrabal‐Gómez et al. (2024)
General brain health	Review	Endurance exercise	Multiple factors: BDNF, irisin, ketone bodies, serotonin, etc.	Exercise supports brain health and may delay age‐related cognitive decline	Jachim et al. (2020)

## 5 Molecular Mechanisms of Exercise‐Induced Neurogenesis

It is important to note that the majority of molecular pathways described above including BDNF, Notch, Wnt, and PI3K/Akt signaling have been characterized primarily in rodent models. Although these mechanisms provide critical biological insight, direct confirmation of their role in adult human hippocampal neurogenesis remains limited.

### 5.1 BDNF Pathway

**FIGURE 1:**
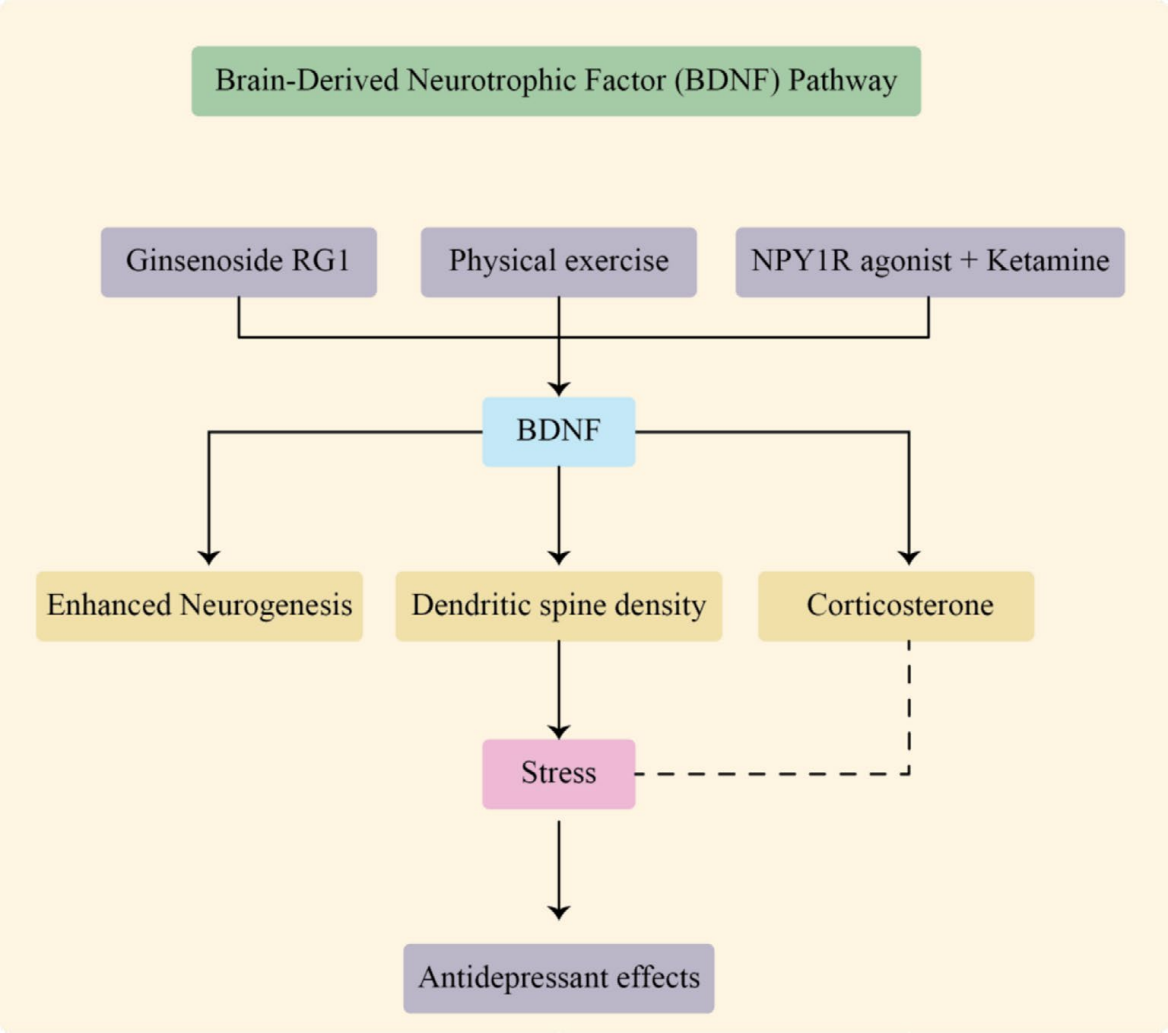
Exercise‐induced activation of the BDNF signaling pathway promotes adult hippocampal neurogenesis and cognitive function. This figure illustrates the central role of brain‐derived neurotrophic factor (BDNF) in mediating the neurogenic and cognitive benefits of physical exercise. Aerobic, resistance, and high‐intensity interval training (HIIT) increase neuronal activity and metabolic demand in the hippocampus, leading to enhanced expression and release of BDNF. BDNF binds to its high‐affinity receptor, tropomyosin receptor kinase B (TrkB), triggering downstream signaling cascades including the PI3K/Akt, MAPK/ERK, and PLCγ pathways. Activation of these pathways promotes neural stem cell (NSC) proliferation, neuronal differentiation, dendritic growth, synaptic plasticity, and neuronal survival within the subgranular zone (SGZ) of the dentate gyrus. Exercise‐induced myokines, such as irisin (derived from FNDC5) and cathepsin B, further contribute to BDNF upregulation and neuroplasticity. Collectively, these molecular events enhance adult hippocampal neurogenesis, improve learning and memory, and increase resilience against age‐related cognitive decline and neurodegenerative processes.

BDNF is one of the most extensively studied molecules involved in regulating adult hippocampal neurogenesis. It plays a critical role in neuronal survival, synaptic plasticity, and cognitive and emotional function. Physical exercise is known to enhance BDNF expression, which in turn supports neurogenesis and improves brain resilience (as shown in Figure 1).

Jachim et al. (2020) showed that sustained aerobic exercise upregulates several neurotrophic factors, including BDNF, FNDC5/irisin, and cathepsin B. Their study highlighted that this energetic stress response, triggered by endurance training, enhances neuroplasticity and supports brain repair, particularly in aging and neurodegenerative conditions (Jachim et al. 2020). Jiang et al. (2012) demonstrated that Ginsenoside Rg1, a compound found in *Panax ginseng* , exerts antidepressant‐like effects by activating the BDNF signaling pathway. In their study, Rg1 increased BDNF expression in the hippocampus, promoting neurogenesis and enhancing dendritic spine density. Interestingly, this effect occurred independently of traditional monoamine neurotransmitter pathways, suggesting a direct neurotrophic mechanism. Rg1 also mitigated stress‐induced elevations in corticosterone, which are known to impair neuroplasticity (Jiang et al. 2012).

The influence of exercise intensity on BDNF and neurogenesis was further explored by Okamoto et al. (2021), who compared HIIT and moderate‐intensity continuous training (MICT) in rats. Both forms of exercise significantly increased hippocampal BDNF levels and improved spatial memory, with HIIT offering comparable benefits in a shorter time frame. This suggests that even time‐efficient exercise regimens can effectively promote neurogenesis through BDNF upregulation (Okamoto et al. 2021). Together, these studies reinforce the central role of BDNF in mediating exercise‐ and treatment‐induced neurogenesis. Whether through lifestyle interventions such as aerobic training, natural compounds like ginsenosides, or pharmacological combinations, enhancing BDNF signaling offers a promising strategy for improving cognitive function and emotional resilience. Further research is warranted to refine these approaches and explore their clinical application in neurodegenerative and psychiatric conditions.

### 5.2 Notch Signaling Pathway

The Notch signaling pathway plays a vital role in regulating neural stem cell maintenance, differentiation, and neurogenesis. Its activity declines with age and has been linked to impaired brain plasticity in neurodegenerative and stress‐related conditions. Recent studies suggest that physical exercise may positively modulate this pathway, thereby supporting neuronal regeneration and cognitive health.

**FIGURE 2:**
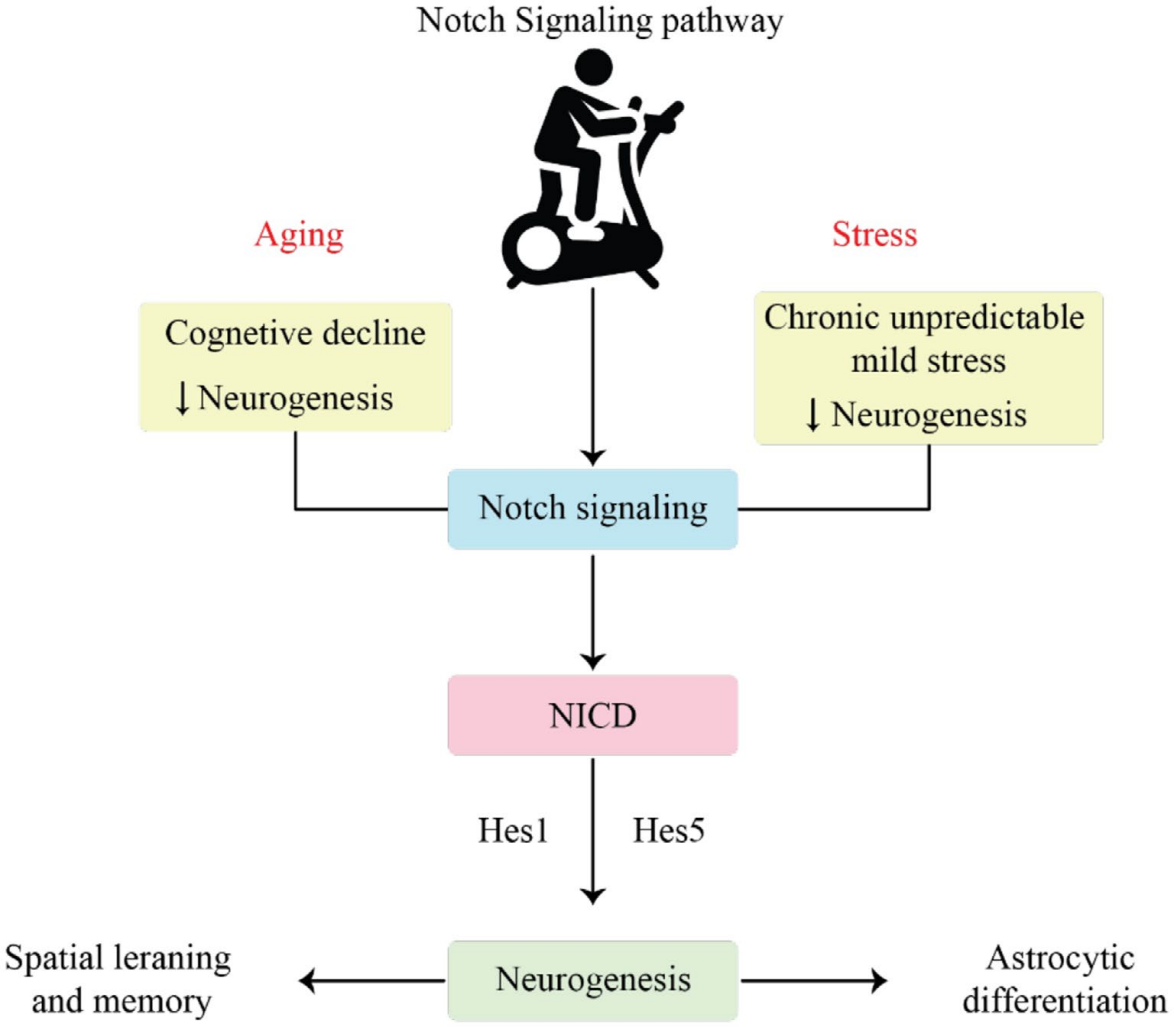
Modulation of Notch signaling by physical exercise supports neural stem cell maintenance and neurogenesis during aging. This figure depicts the regulatory role of the Notch signaling pathway in exercise‐induced hippocampal neurogenesis, particularly in aging and disease contexts. Under physiological conditions, activation of the Notch receptor by ligands such as Delta‐like and Jagged leads to cleavage of the Notch intracellular domain (NICD), which translocates to the nucleus and induces transcription of target genes including Hes1 and Hes5. These genes are essential for maintaining the neural stem cell pool, regulating cell fate decisions, and preventing premature differentiation. Aging, chronic stress, ischemic injury, and neurodegenerative diseases are associated with downregulation of Notch signaling, resulting in impaired neurogenesis and cognitive dysfunction. Regular physical exercise restores Notch pathway activity by increasing NICD levels, enhancing Hes1/Hes5 expression, and promoting autophagy‐related mechanisms. This restoration supports balanced NSC proliferation and differentiation, reduces aberrant astrocytic fate commitment, and improves hippocampal‐dependent learning and memory.

Chen et al. (2025) examined the effects of different exercise modalities on cognitive function and hippocampal neurogenesis in naturally aging rats. Aging was associated with reduced expression of neurogenic markers (DCX, Ki67, and GFAP), CI, and accumulation of Alzheimer's‐related proteins such as APP and Aβ. The researchers also observed a significant downregulation of Notch signaling and autophagy markers in the hippocampus. However, a 12‐week regimen of aerobic, resistance, or combined exercise significantly upregulated Notch signaling and its downstream targets, including Hes1 and Hes5. Exercise also restored autophagy‐related proteins such as LC3, Beclin1, and p62. These molecular changes were accompanied by enhanced neurogenesis and improved spatial learning and memory, suggesting that exercise mitigates age‐related cognitive decline through Notch pathway activation (Chen et al. 2025). Further evidence for the importance of Notch signaling comes from a study by Guo et al. (2009), who investigated its role in neurogenesis following ischemic injury and chronic stress. In adult rats exposed to chronic unpredictable mild stress after ischemia, behavioral signs of depression were accompanied by reduced hippocampal neurogenesis. The researchers found decreased expression of the Notch1 intracellular domain (NICD) and downstream effectors Hes1 and Hes5, along with suppressed cell proliferation in the SGZ. Although Hes1 and Hes5 partially recovered by Day 28 post‐injury, NICD levels remained low, and neurogenesis was impaired. Notably, astrocytic differentiation increased, indicating a shift in neural stem cell fate. These findings suggest that disrupted Notch1 signaling contributes to neurogenic deficits in response to stress and ischemic damage (Guo et al. 2009). These changes were accompanied by improved memory and increased hippocampal neurogenesis, suggesting that exercise mitigates age‐related cognitive decline through Notch pathway activation (Figure 2). Together, these studies underscore the central role of the Notch pathway in adult neurogenesis and cognitive function. Although age, stress, and injury can disrupt this signaling cascade, physical exercise appears to counteract these effects by restoring Notch activity and promoting neural repair. Targeting Notch signaling, through behavioral or pharmacological interventions, may offer a promising strategy for enhancing neurogenesis and protecting cognitive health in aging and disease.

### 5.3 Wnt Signaling Pathway

**FIGURE 3:**
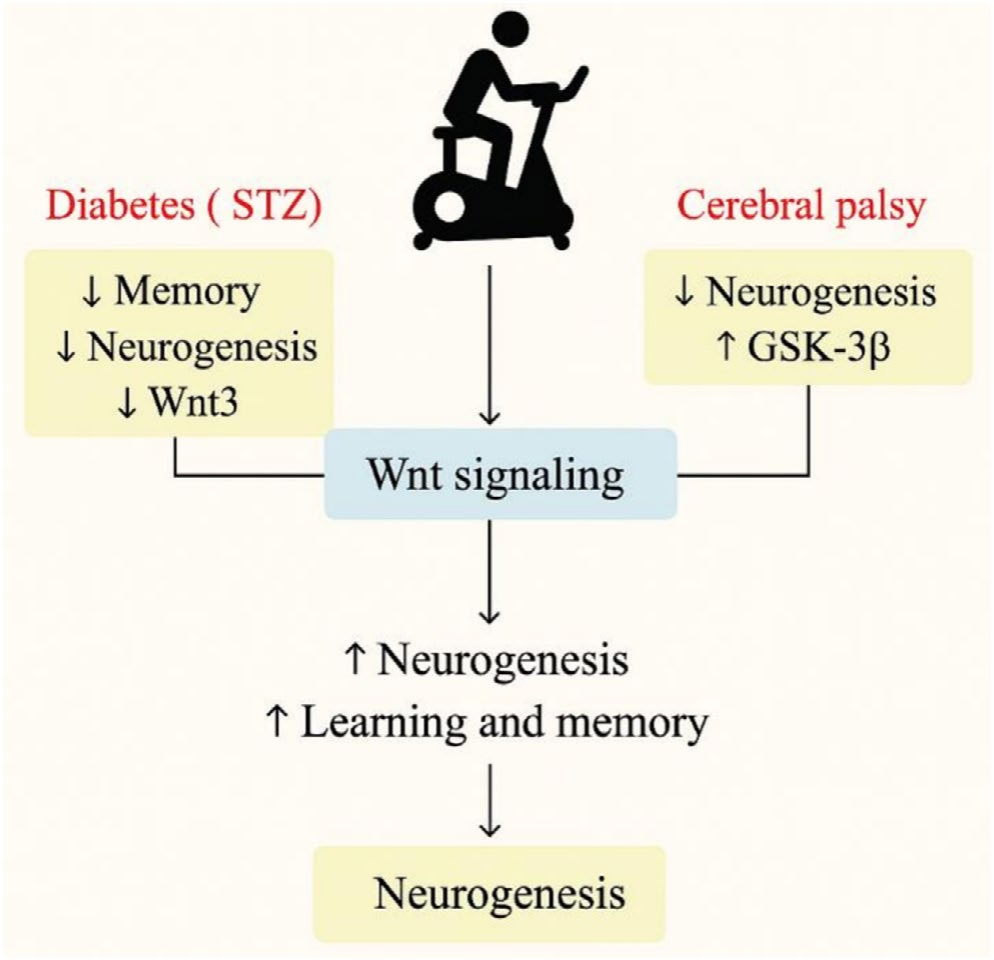
Exercise‐mediated activation of Wnt signaling enhances hippocampal neurogenesis and synaptic plasticity. This figure summarizes the role of Wnt signaling in mediating the pro‐neurogenic effects of physical exercise in the hippocampus. Exercise stimulates the expression of Wnt ligands (e.g., Wnt3), which activate the canonical Wnt/β‐catenin pathway in neural progenitor cells. This activation inhibits glycogen synthase kinase‐3β (GSK‐3β), preventing β‐catenin degradation and allowing its nuclear translocation. Nuclear β‐catenin regulates transcription of genes involved in cell proliferation, neuronal differentiation, and synaptic development. Exercise‐induced activation of upstream signaling pathways, including PI3K/Akt, further stabilizes β‐catenin and enhances Wnt signaling efficacy. In pathological conditions such as diabetes‐associated cognitive impairment, Alzheimer's disease, and neurodevelopmental disorders, suppression of Wnt signaling contributes to reduced neurogenesis and cognitive deficits. Physical exercise counteracts these effects by restoring Wnt pathway activity, increasing the number of BrdU‐ and DCX‐positive cells in the dentate gyrus, enhancing synaptic plasticity, and improving spatial learning and memory.

The Wnt signaling pathway is essential for regulating adult neurogenesis, synaptic plasticity, and cognitive function. Disruptions in this pathway are associated with various neurological disorders, including diabetes‐related cognitive decline and cerebral palsy. Emerging evidence suggests that physical exercise can modulate Wnt signaling, thereby improving memory, learning, and neuronal regeneration (Figure 3). Kim et al. (2016) investigated the effects of treadmill exercise on cognitive function in a rat model of AD combined with diabetes. Diabetes was induced using streptozotocin (STZ), resulting in impairments in short‐term memory and spatial learning. Behavioral assessments using the step‐down avoidance and 8‐arm radial maze tests confirmed cognitive deficits. Immunohistochemical analysis showed reduced hippocampal neurogenesis, with fewer BrdU‐positive and DCX‐positive cells, alongside decreased Wnt3 expression and increased glycogen synthase kinase‐3β (GSK‐3β) activity—indicating suppression of the Wnt pathway. A 12‐week treadmill exercise intervention reversed these effects by improving memory, increasing neurogenesis, upregulating Wnt3, and inhibiting GSK‐3β. These results suggest that exercise restores Wnt pathway activity, supporting cognitive function and neuronal growth in diabetic conditions (Kim et al. 2016). Building on these findings, Cho et al. (2018) examined the role of the PI3K‐Akt‐Wnt signaling axis in a cerebral palsy rat model induced by maternal lipopolysaccharide (LPS) injection. Beginning at 5 weeks of age, the affected offspring underwent 6 weeks of treadmill training. Exercise significantly improved short‐term memory, increased hippocampal cell proliferation, and reduced neuronal apoptosis. Molecular analyses revealed enhanced activation of the PI3K‐Akt pathway, which closely interacts with Wnt signaling. Exercise elevated Wnt expression and concurrently inhibited GSK‐3β activity and β‐catenin degradation, facilitating improved neurogenesis and synaptic stability. These findings reinforce the view that Wnt pathway modulation plays a critical role in exercise‐induced cognitive improvements in neurodevelopmental disorders (Cho et al. 2018). Thus, these studies highlight Wnt signaling as a key mechanism through which exercise promotes neurogenesis and cognitive resilience. By regulating key molecular targets—such as Wnt3, GSK‐3β, and β‐catenin—exercise enhances synaptic plasticity and supports neuronal survival. These findings underscore the therapeutic potential of targeting the Wnt pathway through lifestyle interventions like physical activity in both neurodegenerative and neurodevelopmental contexts.

### 5.4 PI3K/Akt Pathway

The phosphatidylinositol 3‐kinase (PI3K)/Akt signaling pathway plays a central role in regulating neurogenesis, synaptic plasticity, and neuronal survival. This pathway is especially relevant in neurological and psychiatric conditions such as cerebral palsy, post‐traumatic stress disorder (PTSD), and Huntington's disease (HD), where cognitive dysfunction is common. Physical exercise has been shown to activate PI3K/Akt signaling, promoting hippocampal neurogenesis and improving brain function across various models. In a PTSD mouse model, Sun et al. (2020) explored the contribution of Akt signaling to exercise‐induced neurogenesis. Treadmill training significantly promoted the proliferation and differentiation of hippocampal NSCs, improving cognitive and emotional outcomes associated with PTSD. However, when Akt activity was pharmacologically inhibited using GSK690693, these beneficial effects were lost, confirming that Akt activation is essential for exercise‐related improvements in neurogenesis and behavior (Sun et al. 2020).

Similarly, the importance of Akt signaling in neurodegenerative conditions was highlighted in a study on the R6/1 mouse model of HD. The researchers found impaired basal and exercise‐induced neurogenesis, which was linked to reduced Akt phosphorylation in the hippocampus. Although female R6/1 mice showed modest improvements in neurogenesis following voluntary running and environmental enrichment, the enhancements were significantly lower than in wild‐type controls. The study further indicated that the neurogenic response to exercise was compromised by deficient Akt pathway activation, suggesting a mechanistic link between Akt dysfunction and impaired neurogenesis in HD (Ransome and Hannan 2013). Collectively, these studies underscore the critical role of the PI3K/Akt pathway in supporting hippocampal neurogenesis, neuronal survival, and cognitive function. Exercise appears to enhance this pathway's activity, offering neuroprotective benefits across a range of neurological and psychiatric disorders. Targeting PI3K/Akt signaling, through physical activity or adjunctive therapies, may hold therapeutic promise for mitigating cognitive deficits and enhancing brain resilience in both developmental and degenerative conditions.

### 5.5 Other Relevant Pathways

In addition to well‐characterized pathways such as BDNF and PI3K/Akt, several other signaling cascades, including the mitogen‐activated protein kinase (MAPK), VEGF, and glucocorticoid pathways, contribute to the neurogenic and cognitive benefits of physical exercise. These mechanisms play diverse roles in regulating brain plasticity, vascular integrity, and stress responses. The MAPK pathway is essential for neuroplasticity and cellular resilience, especially in the context of neurodegenerative diseases like AD. Sun et al. (2018) evaluated the impact of treadmill exercise in a rat model of AD induced by hippocampal injection of amyloid‐beta (Aβ). As expected, Aβ administration impaired spatial memory and induced neuroinflammation. However, regular treadmill exercise significantly improved cognitive performance, reduced inflammation in the dentate gyrus, and promoted hippocampal neurogenesis. Molecular analysis showed that exercise modulated MAPK signaling by altering the activity of key proteins, including ERK, JNK, and p38 MAPK. These results suggest that the MAPK pathway mediates exercise‐induced neuroprotection and may be a viable target in AD therapy (Sun et al. 2018). Vascular endothelial growth factor (VEGF) is another important mediator, known for its role in angiogenesis and neurogenesis within the hippocampus. Kiuchi et al. (2012) investigated how VEGF contributes to the antidepressant effects of exercise in mice exposed to chronic stress. Prolonged stress led to depressive behaviors, reduced neurogenesis, and decreased microvascular density in the dentate gyrus. Regular physical activity reversed these deficits by promoting neuronal proliferation and enhancing hippocampal blood vessel density. Importantly, when VEGF receptor signaling (Flk‐1) was inhibited, these benefits were abolished. This highlights VEGF as a key factor in exercise‐induced stress resilience and brain remodeling (Kiuchi et al. 2012).

Glucocorticoid signaling, particularly through corticosterone in rodents, also plays a significant role in exercise‐mediated neurogenesis. Chang et al. (2008) examined how treadmill exercise influences hippocampal plasticity via the glucocorticoid pathway. A 5‐week training program increased the number of DCX‐positive neuronal progenitor cells in the hippocampus, indicating enhanced neurogenesis. This effect was accompanied by a transient rise in corticosterone levels and a reduction in mineralocorticoid receptor expression. Interestingly, surgical adrenalectomy, which lowers circulating corticosterone, reduced the neurogenic response to exercise. Additionally, pharmacological blockade of MR using spironolactone further enhanced neurogenesis. These findings suggest that MR downregulation, rather than total suppression of glucocorticoids, is a key mechanism through which exercise promotes neuronal survival and differentiation (Chang et al. 2008). Together, these studies illustrate that multiple molecular pathways, beyond BDNF and PI3K/Akt, are involved in exercise‐induced neurogenesis. MAPK signaling contributes to neuroinflammation control and synaptic remodeling; VEGF supports vascular and neuronal growth; and glucocorticoid signaling modulates neurogenic responses to stress. Understanding how these diverse systems interact offers deeper insight into the multifactorial benefits of exercise on brain health and supports its role as a therapeutic strategy across a wide range of neuropsychiatric and neurodegenerative conditions.

## 6 Exercise Modalities and Their Effects on Neurogenesis

### 6.1 Aerobic and Endurance Exercise

Aerobic and endurance exercises have been widely recognized for their beneficial effects on neurogenesis, synaptic plasticity, and cognitive function. These forms of physical activity engage multiple molecular pathways that support neuronal survival, brain adaptability, and long‐term cognitive health. Recent studies have provided valuable insights into how different exercise intensities and durations influence brain outcomes. Jang (2020) investigated how prolonged endurance exercise affects autophagy in the hippocampus, a brain region essential for memory and learning. In a mouse model, sustained physical activity led to increased expression of autophagy‐related proteins such as LC3 II, BECLIN1, and ATG7, alongside activation of anabolic signaling through the AKT–mTOR–S6K pathway. Interestingly, despite a decrease in traditional neurotrophic factors like BDNF and NGF, neurogenesis was maintained through upregulation of neuregulin‐1, a protein linked to synaptic plasticity. This suggests that endurance exercise may support neurogenesis via alternative signaling mechanisms, enhancing brain resilience even when classical neurotrophic support is reduced (Jang 2020). The role of exercise intensity in modulating neurogenesis was examined by Inoue et al. (2015), who compared mild (ME) and intense exercise (IE) using a six‐week treadmill training protocol in rats. Moderate‐intensity exercise, performed below the lactate threshold, significantly improved spatial memory and promoted neuronal survival. In contrast, high‐intensity training led to elevated corticosterone levels (an indicator of physiological stress) that negatively affected hippocampal plasticity. Transcriptomic analysis revealed that ME was associated with beneficial regulation of genes involved in lipid metabolism, protein synthesis, and inflammation, whereas IE triggered excessive immune activation that may limit the cognitive benefits of neurogenesis. These findings highlight the importance of exercise intensity in optimizing brain outcomes (Inoue et al. 2015). Thus, these studies highlight the complex interplay between exercise modality, intensity, and molecular signaling in shaping hippocampal function. Endurance and aerobic exercises enhance neurogenesis through diverse mechanisms, including autophagy modulation, stress hormone regulation, and neurotrophic signaling. Importantly, moderate‐intensity and interval‐based regimens appear particularly effective in balancing physiological stress with neuroprotective gains. Understanding these nuances is critical for designing exercise‐based interventions aimed at preventing cognitive decline and promoting brain health across the lifespan.

### 6.2 Resistance Training

Although the cognitive benefits of aerobic exercise are well‐documented, growing evidence suggests that resistance training also plays a meaningful role in supporting brain health. In particular, resistance exercise appears to influence molecular pathways involved in neurogenesis and synaptic plasticity, especially within the hippocampus—a region critical for learning and memory. Suijo et al. (2013) explored the effects of progressive resistance wheel training in mice over a two‐week period. The study assessed changes in molecular markers related to neuroplasticity, including BDN and cyclic AMP response element‐binding protein (CREB), both of which are central to learning, memory, and synaptic strengthening. Behavioral testing using the Morris water maze confirmed that resistance training improved spatial learning and memory. Concurrently, significant increases in hippocampal BDNF and CREB expression were observed, suggesting that resistance exercise enhances cognitive performance by activating key neuroplasticity‐related pathways. In addition to its central effects, resistance training also activated the mammalian target of rapamycin (mTOR) pathway in peripheral muscle tissue, a key regulator of protein synthesis and muscular adaptation. Notably, the levels of phosphorylated mTOR and p70S6K in the soleus muscle were positively correlated with hippocampal BDNF expression. This finding suggests a mechanistic link between skeletal muscle activity and brain‐derived neurotrophic signaling, highlighting how peripheral adaptations may influence central nervous system plasticity (Suijo et al. 2013).

### 6.3 HIIT

HIIT has gained recognition as a time‐efficient exercise strategy that delivers both physical and cognitive benefits. Unlike continuous endurance training, HIIT involves repeated short bursts of intense effort followed by brief recovery periods, providing comparable or even superior outcomes in less time. Okamoto et al. (2021) investigated the effects of HIIT on hippocampal neurogenesis and spatial memory in adult male Wistar rats. The study compared HIIT with MICT, evaluating both cognitive outcomes and molecular adaptations. The HIIT protocol consisted of short sprints at 60 m/min, whereas the MICT group ran continuously at 20 m/min. After 4 weeks of training, animals in the HIIT group demonstrated improved exercise capacity, as measured by performance in an incremental running test. Both HIIT and MICT enhanced spatial memory and increased AHN. However, HIIT produced greater metabolic adaptations, as evidenced by elevated citrate synthase activity in the plantaris muscle—a marker of mitochondrial function. Importantly, both exercise modalities were associated with increased levels of BDNF, a key regulator of neuroplasticity, learning, and memory. These findings suggest that HIIT is not only efficient but also effective in promoting brain health. By enhancing BDNF signaling and supporting neurogenesis, HIIT emerges as a viable alternative to traditional endurance exercise—offering cognitive and physical benefits within a shorter time frame (Okamoto et al. 2021).

## 7 Influence of Dietary Polyphenols on Neurogenesis and Brain Health During Aging

**FIGURE 4:**
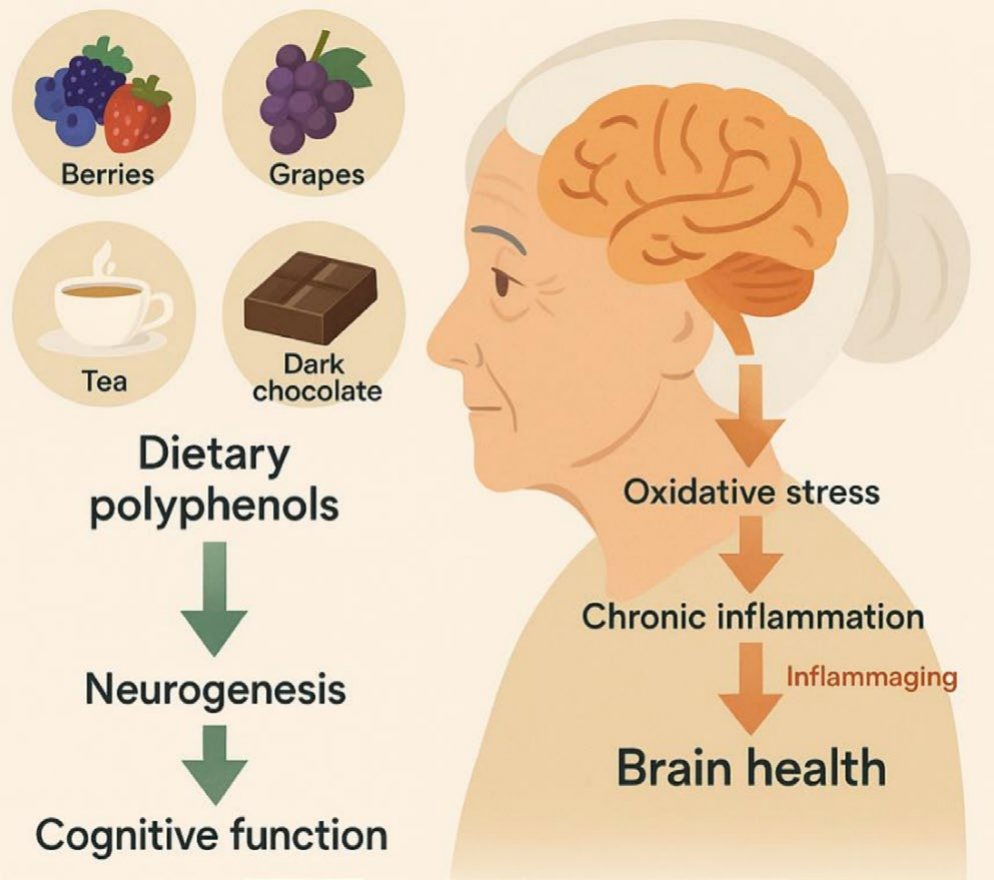
Role of dietary polyphenol–rich foods in modulating oxidative stress, inflammation, and cognitive function during aging. This schematic illustrates the contribution of common polyphenol‐rich foods, including berries, grapes, tea, and dark chocolate, to the maintenance of brain health in aging. Dietary polyphenols derived from these foods support hippocampal neurogenesis and cognitive function through antioxidant and anti‐inflammatory mechanisms. Aging is associated with increased oxidative stress and chronic low‐grade inflammation (“inflammaging”), which negatively impact neuronal integrity and cognitive performance. Regular intake of polyphenol‐rich foods may counteract these age‐related processes by reducing oxidative damage and inflammatory signaling, thereby promoting neuroplasticity and preserving cognitive function. This figure highlights the translational relevance of dietary choices as accessible, non‐pharmacological strategies to support brain health and mitigate cognitive decline in older adults.

As the global population ages, the need for effective strategies to preserve cognitive function and delay brain aging is becoming increasingly urgent. A major contributor to age‐related cognitive decline is the progressive reduction in adult neurogenesis (AN), largely due to cellular senescence and changes in the neurogenic microenvironment. This decline is closely associated with elevated oxidative stress and chronic low‐grade inflammation, collectively termed inflammaging, which disrupts neural function and increases susceptibility to neurodegenerative diseases (Carpentier and Palmer 2009; Chen et al. 2012; Glass et al. 2010; Grabska‐Kobyłecka et al. 2023; Sarubbo et al. 2018; Silva and Pogačnik 2020). Polyphenols, a diverse group of bioactive compounds found in plant‐based foods such as berries, grapes, and tea, have received growing attention for their potential to support brain health during aging. Their ability to cross the blood–brain barrier and accumulate in neural tissues makes them attractive candidates for neuroprotection (Liu 2022; Zhang et al. 2013). Although traditionally recognized for their antioxidant and anti‐inflammatory properties, polyphenols also modulate intracellular signaling pathways involved in neural cell survival and plasticity. These include the PI3K/Akt, ERK, and Wnt pathways, as well as key transcription factors such as SIRT1, Nrf2, and NF‐κB, all of which contribute to maintaining neural stem cell viability and reducing neuroinflammation (le Tang et al. 2021; Longo and Massa 2013; Moosavi et al. 2015) (Figure 4).

Specific polyphenolic compounds, such as resveratrol, curcumin, quercetin, and epigallocatechin gallate (EGCG), have demonstrated the ability to enhance synaptic plasticity and improve memory in animal models of neurodegeneration (Karuppagounder et al. 2013; Suganuma et al. 1998). Some polyphenols, such as 7,8‐dihydroxyflavone, can even mimic BDNF by activating TrkB receptors, thereby promoting neuronal survival and growth (Tangsaengvit et al. 2013). Others, including hesperetin and naringenin, act indirectly by modulating oxidative and inflammatory mediators, contributing to neuronal repair and resilience (Youdim et al. 2003). Importantly, these neuroprotective effects are not limited to disease treatment. Regular intake of polyphenol‐rich foods or supplements has shown potential in delaying the onset or reducing the risk of neurodegenerative conditions such as Alzheimer's and Parkinson's diseases (Granzotto and Zatta 2014; Mandel et al. 2006; Rossi et al. 2008). Animal studies and early‐phase human trials suggest that polyphenols help prevent the accumulation of pathological proteins, support mitochondrial function, and enhance cerebral blood flow—factors that collectively contribute to better cognitive performance (Casadesus et al. 2004). Polyphenols may also have mood‐stabilizing properties. Several studies indicate that they can alleviate symptoms of depression and anxiety by increasing levels of monoamines such as serotonin, dopamine, and norepinephrine, partly through inhibition of monoamine oxidase (MAO) enzymes. These neuromodulatory effects further enhance neuroplasticity and emotional regulation, suggesting broader mental health benefits (Dias et al. 2012; Ito et al. 2008; Mattova et al. 2023; Vignes et al. 2006; Zhang et al. 2019). Clinical evidence also supports the cognitive benefits of polyphenols. Interventions using polyphenol‐rich juices or extracts, such as pomegranate, grape, or cherry juice, have led to measurable improvements in memory and cognitive performance in older adults, even over relatively short durations. These findings reinforce the value of polyphenol‐rich diets as part of a broader strategy to maintain brain health during aging (Kean et al. 2015; Rajaram et al. 2019; Valls‐Pedret et al. 2012).

## 8 Synergistic Effects of Polyphenols and Exercise on Hippocampal Health

**FIGURE 5:**
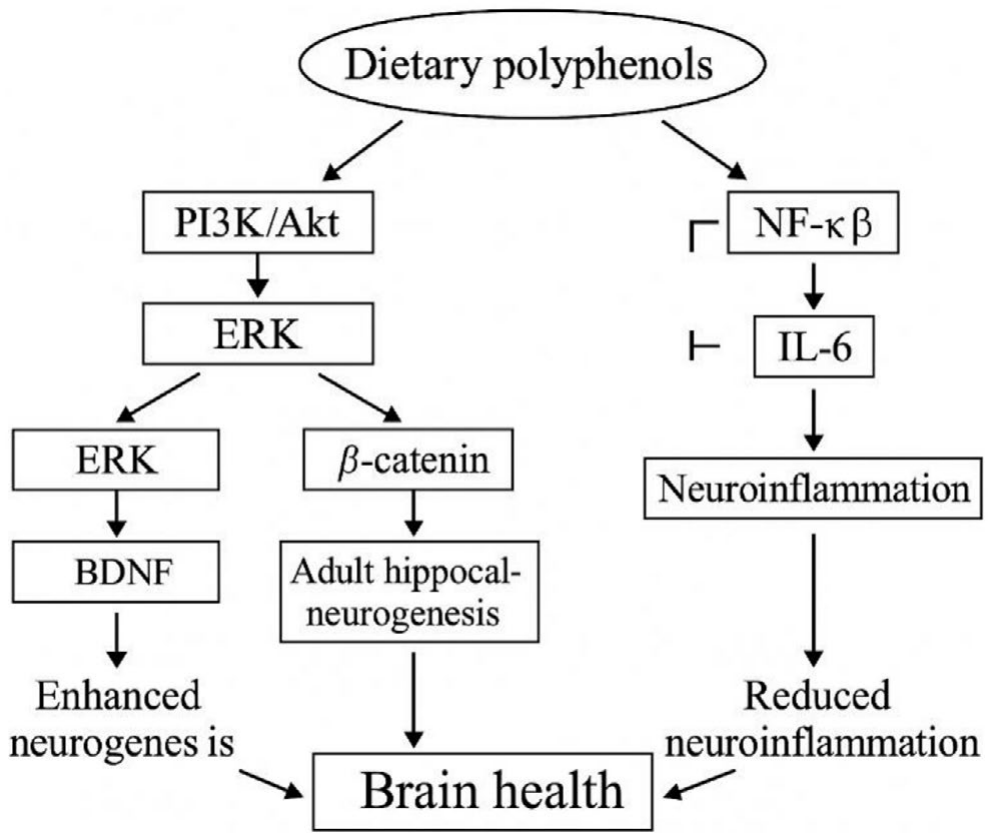
Molecular mechanisms by which dietary polyphenols modulate hippocampal neurogenesis and neuroinflammation to support brain health. This schematic illustrates the proposed signaling pathways through which dietary polyphenols influence hippocampal function and cognitive health. Polyphenols activate the PI3K/Akt and ERK signaling cascades, leading to increased expression of brain‐derived neurotrophic factor (BDNF) and activation of β‐catenin–dependent pathways, both of which promote adult hippocampal neurogenesis. In parallel, dietary polyphenols inhibit pro‐inflammatory signaling by suppressing nuclear factor kappa B (NF‐κB) activation and reducing the expression of inflammatory mediators such as interleukin‐6 (IL‐6), thereby attenuating neuroinflammation. The combined enhancement of neurogenesis and reduction of neuroinflammatory processes converge to support hippocampal integrity and overall brain health. This figure provides an integrative mechanistic framework linking diet‐derived bioactive compounds to molecular pathways relevant to cognitive preservation during aging and neurodegenerative conditions.

The synergistic effects of combining polyphenolic supplementation with structured physical activity have been widely observed across preclinical and clinical studies. Numerous investigations have demonstrated that these dual interventions not only enhance hippocampal neurogenesis but also modulate key molecular pathways, such as BDNF, SIRT1, AMPK, and Wnt, whereas attenuating oxidative stress and inflammation (as shown in Figure 5). In humans, clinical evidence predominantly supports improvements in cognitive performance and neurotrophic biomarkers, whereas direct assessment of hippocampal neurogenesis remains technically challenging. As a result, translational conclusions are often inferred from converging behavioral, biochemical, and neuroimaging outcomes rather than direct cellular measurements.

These interactions are summarized in Table 2, which provides an overview of disease models, treatment conditions, mechanisms, and outcomes across various polyphenol‐exercise combinations.

**TABLE 2:** Combined effects of polyphenols and exercise on neurogenesis and brain function.

Disease/condition	Type of study	Treatment condition	Mechanisms investigated	Results	References
Type 2 diabetes‐induced cognitive dysfunction	Pre‐clinical	Curcumin + exercise	↓ ER stress (BiP, CHOP), ↓ IL6, TNFα, IL10	↑ Memory retention, ↓ weight, improved glucose/lipid profile	Cho et al. (2020)
	Pre‐clinical	Saffron + resistance training	↑ BDNF	Combo improved glucose metabolism and neuroplasticity	Valipour Dehnou (2024)
Epilepsy (pilocarpine‐induced seizures)	Pre‐clinical	Curcumin + exercise	↓ MAO & AChE, ↑ BDNF mRNA, ↓ ROS, ↑ thiols	↓ Seizure frequency, improved neuronal markers	Ogunsuyi et al. (2023)
Depression (chronic stress)	Pre‐clinical	Curcumin + exercise	↑ Cell survival in CA3, ↓ depressive‐like behavior	↓ Immobility, ↑ locomotor activity, neuroprotection in hippocampus	Ahmadi et al. (2024)
	Pre‐clinical	Quercetin + exercise	↓ Inflammation, ↑ serotonin, ↑ mitophagy proteins (PINK1, Parkin)	Restored behavior and hippocampal integrity	Abdallah et al. (2024)
Lead‐induced neurotoxicity	Pre‐clinical	Curcumin + exercise	↑ BDNF, ↓ MDA, ↑ antioxidant capacity	Reduced oxidative stress and hippocampal damage	Hosseinzadeh et al. (2013)
	Pre‐clinical	HIIT + curcumin	↑ SOD & CAT, ↓ lead accumulation	Improved motor/cognitive function, ↓ oxidative stress	Noruzi et al. (2023)
	Pre‐clinical	Curcumin + exercise	↑ BDNF, ↓ TBARS	Neuroprotection in cerebellum	Habibian et al. (2016)
Arsenic‐induced brain injury	Pre‐clinical	HIIT + curcumin	↓ Caspase‐3, ↓ GRP78, ↑ TAC	Curcumin protective, HIIT alone worsened damage	Hosseinlou et al. (2020)
Cerebral ischemia	Pre‐clinical	Curcumin vs. HIIT	Neuronal density, behavioral tests	Curcumin superior to HIIT alone	Dias et al. (2012)
Morphine‐induced cognitive impairment	Pre‐clinical	Curcumin + exercise	Curcumin counteracted DMSO effects	Improved memory, reversed morphine‐induced deficits	Elhampour et al. (2019)
Alzheimer's disease	Pre‐clinical	Chronic exercise + resveratrol	↓ Aβ, inflammation, apoptosis, ↑ synaptic proteins	Synergistic neuroprotection	Broderick et al. (2020)
	Pre‐clinical	Resveratrol + aerobic exercise	AMPK/PGC‐1α/SIRT1 axis	Reversed AD‐induced molecular decline	Rashet et al. (2024)
	Pre‐clinical	Resveratrol + aerobic exercise	Ferroptosis, GSH, GPx4, Nrf2, HO‐1	↓ Ferroptosis, ↑ antioxidant defense	Habibi et al. (2023)
	Clinical	Resveratrol supplement (human)	Memory function, hippocampal microstructure	No significant changes; some trend toward memory protection	Huhn et al. (2018)
	Pre‐clinical	Green tea ± exercise	Antioxidant mechanisms (ROS)	Green tea improved redox status but not memory	Flôres et al. (2014)
	Pre‐clinical	Quercetin + exercise	↓ Oxidative stress, ↑ antioxidant enzymes	Enhanced spatial memory, neuroprotection	Molaei et al. (2020)
	Pre‐clinical	Saffron + endurance training	↑ PGC1‐α	Enhanced mitochondrial biogenesis in hippocampus	Azarian et al. (2020)
	Pre‐clinical	Saffron + aerobic exercise	↓ Tau accumulation	Improved spatial memory, reduced tau pathology	Bazyar Halimehjani and Shabani (2023)
Chronic fatigue syndrome	Pre‐clinical	Resveratrol alone	↑ Neurogenesis, ↓ apoptosis, ↑ BDNF, acetyl‐p53	Improved hippocampal plasticity	Moriya et al. (2011)
Exercise‐induced oxidative stress	Pre‐clinical	Endurance exercise ± resveratrol	↓ 8‐OHdG, ↓ carbonyls, CPK, LDH	↓ Oxidative stress; no effect on muscle injury markers	Vafaee et al. (2019)
	Clinical	Green tea + exercise	Anaerobic performance, fatigue	↑ Anaerobic power; most benefit in females	Mao and Thanaphonganan (2024)
H_2_O_2_‐induced oxidative stress	Pre‐clinical	Curcumin + HIIT	↓ Bax, ↑ Bcl‐2, ↓ Bax/Bcl‐2 ratio	Reduced apoptosis, improved resilience	Toktam‐Barmar et al. (2022)
Colon cancer (cognition)	Pre‐clinical	Quercetin + intermittent exercise	↑ BDNF & CREB	Combined intervention superior for neuroplasticity	Sadeghi et al. (2022)
	Pre‐clinical	Quercetin + exercise	↓ inflammation, ↑ BDNF/TrKβ/β‐catenin	↓ Depressive behavior, improved hippocampal integrity	Sadighparvar et al. (2020)
Chronic unpredictable stress	Pre‐clinical	Crocin (saffron) ± exercise	CA3 neuroprotection	Crocin effective, exercise alone ineffective under stress	Dastgerdi et al. (2018)
Diet‐induced cognitive/anxiety effects	Pre‐clinical	Soy‐deficient diet + exercise	↑ BDNF, ↑ NMDA receptor, ↑ synaptic efficiency	Exercise reversed soy‐deficient memory impairments	Cheng et al. (2018)
	Pre‐clinical	Soy oil vs. hydrogenated fat + exercise	Na^+^/K^+^‐ATPase modulation	Soy + exercise improved cognitive and emotional outcomes	Teixeira et al. (2011)
Age‐related cognitive decline	Clinical trial	Soy peptide + exercise	Neurocognitive support, muscle strength	↑ Memory and muscle strength	Imaoka et al. (2022)
	Clinical trial	Soy peptide + exercise	Executive function, motor performance	↑ Calculation ability and mobility	Imaoka et al. (2019)
	Clinical trial	Grape juice + exercise	↑ BDNF, synaptic plasticity	Memory improved only in combo group	Trevizol et al. (2018)
	Pre‐clinical	EGCG + β‐alanine + exercise	↑ Neurogenesis, ↑ BDNF	Exercise improved cognition; polyphenols alone had no added benefit	Gibbons (2014)
	Pre‐clinical	Resveratrol + HIIT	NAD^+^/NADH, SIRT3/4, AMPK, SOD2	↑ Antioxidant/mitochondrial function, complementary SIRT modulation	Amirazodi et al. (2022)
Cognitive performance in students	Clinical trial	Black mulberry/pumpkin seeds + exercise	↑ BDNF, ↓ glucocorticoid receptor, ↑ GDH	Improved executive function, memory span	Shalan et al. (2021, 2020)
Healthy rats (baseline cognition)	Pre‐clinical	Saffron + endurance training	↑ BDNF, NT‐3, serotonin	Improved short‐term memory, dual central/peripheral benefit	Akbari‐Fakhrabadi et al. (2021)

### 8.1 Curcumin and Exercise: Anti‐Inflammatory and Antioxidant Synergy in Stress and Toxicity Models

Curcumin, the primary bioactive compound in turmeric ( *Curcuma longa* ), is known for its potent antioxidant, anti‐inflammatory, and neuroprotective properties. When combined with physical exercise, curcumin may exert additive or synergistic effects on hippocampal function, especially under conditions of chronic stress, metabolic dysfunction, and environmental neurotoxicity. A growing body of preclinical research supports this interaction across various models of brain injury and disease. In a study involving diabetic OLETF rats, Cho et al. (2020) demonstrated that combining moderate exercise with dietary curcumin supplementation (5 g/kg) improved spatial memory, reduced body weight, and improved metabolic parameters. Cognitive improvements, assessed via the Morris water maze, were accompanied by reduced levels of pro‐inflammatory cytokines (IL‐6 and TNF‐α) and markers of endoplasmic reticulum (ER) stress in the hippocampus, suggesting that curcumin and exercise jointly modulate systemic and neuronal stress pathways (Cho et al. 2020). In models of epilepsy, Ogunsuyi et al. (2023) showed that treadmill exercise and curcumin administration reduced seizure frequency and oxidative damage in the hippocampus. The combined intervention also normalized the activity of MAO and acetylcholinesterase, whereas restoring hippocampal expression of BDNF, a key mediator of neuroplasticity and neuronal survival (Ogunsuyi et al. 2023). Similar neuroprotective effects were observed in a chronic stress model. Ahmadi et al. (2024) found that curcumin (100 mg/kg/day) combined with treadmill running improved behavioral outcomes in rats subjected to chronic unpredictable stress, as evidenced by enhanced locomotion and reduced immobility in the forced swim test. Histological analysis showed preserved neuronal integrity in the CA3 region of the hippocampus, supporting the role of this combination in mitigating stress‐induced damage (Ahmadi et al. 2024). Curcumin has also demonstrated efficacy against heavy metal neurotoxicity when paired with physical activity. In a lead exposure model, Hosseinzadeh et al. (2013), reported that aerobic training with curcumin restored hippocampal BDNF levels, increased antioxidant enzyme activity, and reduced lipid peroxidation (Hosseinzadeh et al. 2013). Comparable findings were reported in arsenic‐exposed rats, where Hosseinlou et al. (2020) found that while HIIT alone increased apoptotic markers (e.g., caspase‐3), the addition of curcumin reduced hippocampal oxidative stress and suppressed GRP78 expression, a marker of ER stress (Hosseinlou et al. 2020). Further support comes from Noruzi et al. (2023), demonstrating that combining curcumin with HIIT in lead nitrate‐exposed rats improved cognitive and motor performance while reducing oxidative damage and lead accumulation (Noruzi et al. 2023). Elevated levels of catalase (CAT) and superoxide dismutase (SOD) in the hippocampus indicated enhanced antioxidant defenses. Notably, Habibian et al. (2016) extended these findings to the cerebellum, showing increased BDNF and reduced lipid peroxidation under similar conditions (Habibian et al. 2016).

However, not all findings were uniformly positive. Amaral Dias et al. (2019) reported that curcumin alone improved neuronal survival and motor function in a cerebral ischemia model, whereas HIIT exacerbated brain injury. This suggests that high‐intensity exercise may be neurotoxic under certain conditions unless balanced with neuroprotective agents like curcumin (Amaral Dias et al. 2019). Other studies have focused on molecular signaling pathways. Toktam‐Barmar et al. (2022) showed that curcumin and HIIT reduced hippocampal apoptosis by lowering the Bax/Bcl‐2 ratio in a model of hydrogen peroxide‐induced oxidative stress (Toktam‐Barmar et al. 2022). Similarly, Elhampour et al. (2019) found that in morphine‐sensitized mice, even sub‐effective doses of curcumin and exercise synergistically improved spatial learning, though solvent effects (DMSO) confounded results in some groups (Elhampour et al. 2019). Overall, these findings suggest that curcumin, when combined with structured physical exercise, offers broad neuroprotective benefits by modulating oxidative stress, inflammatory signaling, neurotrophic factor expression, and apoptotic pathways. This combination may be particularly effective in protecting hippocampal function under toxic, metabolic, or stress‐related insults, though exercise intensity and curcumin dosing require careful optimization.

### 8.2 Resveratrol and Exercise: Mitochondrial and Neurotrophic Pathway Enhancement

Resveratrol, a polyphenol found in grapes, berries, and red wine, is well‐known for its antioxidant, anti‐inflammatory, and mitochondrial‐regulating properties. Combined with structured physical exercise, resveratrol may enhance hippocampal function through complementary mechanisms involving energy metabolism, sirtuin activation, and neurotrophic support. Evidence from preclinical and limited clinical studies suggests that this combination can target core pathways implicated in aging and neurodegeneration. In a study by Amirazodi et al. (2022), aged rats underwent HIIT combined with resveratrol supplementation. This intervention significantly improved markers of mitochondrial efficiency and oxidative defense, including NAD^+^/NADH ratio, SOD2, and AMPK activity. Interestingly, while HIIT alone increased SIRT3 expression, resveratrol appeared to differentially modulate sirtuin signaling, suggesting complementary roles in regulating mitochondrial dynamics (Amirazodi et al. 2022). Resveratrol has also demonstrated protective effects in models of AD. Broderick et al. (2020) showed that resveratrol reduced amyloid‐beta accumulation, decreased neuroinflammatory markers, and enhanced synaptic protein expression in AD mice. Although exercise alone had some neuroprotective effects, the combination of exercise and resveratrol yielded similar outcomes to resveratrol monotherapy, indicating overlapping or saturable mechanisms (Broderick et al. 2020). Other studies further support this interaction at the molecular level. Rashet et al. (2024) reported that both aerobic training and resveratrol reversed the AD‐induced suppression of the AMPK/PGC‐1α/SIRT1 axis—a central regulator of mitochondrial biogenesis and cellular energy balance (Rashet et al. 2024). Similarly, Habibi et al. (2023) found that the combination mitigated ferroptosis, a type of iron‐dependent cell death associated with Alzheimer's pathology, by modulating glutathione (GSH) levels and upregulating Nrf2 signaling (Habibi et al. 2023).

At the structural and functional level, Aguilar‐Garcia et al. (2024) observed that both interventions improved motor performance and potentially enhanced hippocampal neurogenesis in rats subjected to mechanical brain injury. These outcomes likely stem from combined anti‐inflammatory and regenerative effects (Aguilar‐Garcia et al. 2024). Even in the absence of overt pathology, resveratrol appears to support hippocampal integrity. Moriya et al. (2011) demonstrated that resveratrol improved neurogenesis and reduced apoptosis in a model of chronic fatigue, resulting in larger hippocampal volume and better locomotor function. These effects are thought to be mediated primarily through sirtuin activation and antioxidant pathways (Moriya et al. 2011). Resveratrol has also been shown to modulate exercise‐induced oxidative stress. Vafaee et al. (2019) reported that although resveratrol did not affect markers of muscle damage, it reduced oxidative DNA and protein damage following acute and endurance exercise. This highlights its potential to buffer oxidative challenges associated with physical activity, especially in older or vulnerable populations (Vafaee et al. 2019). However, human findings remain mixed. A clinical trial by Huhn et al. (2018) in older adults found a non‐significant trend toward improved memory and hippocampal connectivity with resveratrol supplementation, indicating a need for larger, longer‐term studies to confirm efficacy in clinical populations (Huhn et al. 2018). Accordingly, the available evidence suggests that resveratrol and physical exercise converge on mitochondrial regulation, oxidative stress reduction, and neurotrophic signaling to promote hippocampal health. Although some overlap in their effects may limit additional benefits under certain conditions, this combination offers a compelling non‐pharmacological strategy to preserve cognitive function and support brain resilience during aging and neurodegenerative processes.

### 8.3 Quercetin and Exercise: Cognitive and Mood Resilience via Neurotrophic and Anti‐Inflammatory Mechanisms

Quercetin is a widely studied flavonoid found in apples, onions, and various berries, known for its strong antioxidant and anti‐inflammatory properties. Its combination with physical exercise appears to produce synergistic benefits for hippocampal function, particularly in models of neurodegeneration, inflammation, and cancer‐related cognitive decline. Together, these interventions target overlapping pathways involved in oxidative stress reduction, neurotrophic signaling, and emotional regulation. In a study by Molaei et al. (2020), rats with STZ‐induced Alzheimer's‐like pathology exhibited spatial memory deficits and hippocampal oxidative stress. Although quercetin or exercise alone partially improved these outcomes, their combination significantly enhanced memory performance and antioxidant enzyme activity, suggesting a synergistic interaction in restoring hippocampal function under neurodegenerative stress (Molaei et al. 2020). Sadeghi et al. (2022) investigated the combined effects of intermittent physical activity and quercetin supplementation in rats with colon cancer. The study focused on key neuroplasticity markers, including BDNF and CREB. Although exercise alone upregulated both markers, the addition of quercetin produced an additive effect, indicating that quercetin may enhance exercise‐induced neurotrophic responses even in non‐neurological disease contexts (Sadeghi et al. 2022). This synergy also extends to emotional health. Sadighparvar et al. (2020) showed that quercetin and physical activity together reduced depressive‐like behavior in rats with colorectal cancer. The combination suppressed inflammatory cytokines and increased BDNF levels in the prefrontal cortex, supporting the hypothesis that quercetin may potentiate the mood‐regulating effects of exercise through neuroinflammatory modulation (Sadighparvar et al. 2020).

A similar pattern was observed in a study by Abdallah et al. (2024) which assessed depressive behaviors and hippocampal health in LPS‐induced inflammation. The dual intervention of quercetin and treadmill running significantly reduced markers of oxidative stress and inflammation while restoring BDNF and serotonin levels. Notably, mitophagy‐related proteins PINK1 and Parkin were also normalized, suggesting that the protective effect of this combination extends to mitochondrial quality control. Histological analysis revealed reduced neuronal damage in the hippocampus, reinforcing the therapeutic potential of this pairing in inflammatory brain conditions (Abdallah et al. 2024). Thus, these studies highlight the promising role of quercetin and structured exercise in maintaining hippocampal integrity and cognitive resilience. By engaging shared biological pathways, particularly those related to oxidative stress, neurotrophic support, and inflammation, the combination may be especially beneficial in settings of chronic disease, neuroinflammation, and psychological stress. These findings support further exploration of quercetin‐exercise co‐interventions as non‐invasive strategies for protecting brain health.

### 8.4 Saffron and Exercise: Neurotrophic and Anti‐Amyloid Potential in Aging and Neurodegeneration

Saffron ( *Crocus sativus* L.), a traditional medicinal plant rich in bioactive compounds such as crocin and safranal, has shown considerable promise in neuroprotection, particularly through antioxidant, anti‐inflammatory, and neurotrophic mechanisms. When paired with physical exercise, saffron may enhance cognitive resilience by promoting hippocampal plasticity, improving mitochondrial function, and reducing pathological protein accumulation—factors commonly implicated in neurodegenerative diseases and stress‐related disorders. In a model of chronic unpredictable stress, Dastgerdi et al. (2018) demonstrated that crocin supplementation significantly improved learning and memory, whereas exercise alone was insufficient under these conditions. However, their combination produced superior cognitive outcomes and greater neuronal preservation in the hippocampus, suggesting a synergistic effect on stress resilience and hippocampal plasticity (Dastgerdi et al. 2018). Further evidence comes from Valipour Dehnou (2024), who evaluated resistance training with saffron in diabetic rats. Although both interventions reduced blood glucose levels, only the combination significantly increased hippocampal BDNF expression—a key neurotrophic factor involved in memory and synaptic function. This highlights saffron's potential to potentiate the neuroplastic effects of exercise under metabolic stress (Valipour Dehnou 2024). Akbari‐Fakhrabadi et al. (2021) extended these findings by showing that saffron combined with endurance training elevated levels of BDNF, serotonin, and neurotrophin‐3 (NT‐3) in both the hippocampus and skeletal muscle. The improvements in short‐term memory performance observed in Wistar rats suggest that this dual intervention supports neuroplasticity through both central and peripheral pathways (Akbari‐Fakhrabadi et al. 2021).

In AD models, saffron has shown particular value when paired with physical activity. Azarian et al. (2020) found that this combination significantly increased hippocampal PGC‐1α expression, a key regulator of mitochondrial biogenesis and energy metabolism—both of which are often impaired in AD. These changes were associated with better cognitive outcomes, reinforcing the role of mitochondrial support in neurodegenerative resilience (Azarian et al. 2020). Bazyar Halimehjani and Shabani (2023) further demonstrated that the combined use of saffron and aerobic exercise reduced tau protein accumulation, a hallmark of AD pathology, whereas improving spatial memory. These findings point to the potential for saffron and exercise to act on both cognitive function and neuropathological markers, offering a dual protective mechanism (Bazyar Halimehjani and Shabani 2023). These studies underscore the potential of combining saffron supplementation with physical activity as a non‐pharmacological approach to promote hippocampal health. By enhancing neurotrophic factor expression, modulating mitochondrial and synaptic pathways, and reducing disease‐related protein aggregation, this integrated strategy may offer significant cognitive and neuroprotective benefits in aging and neurodegenerative conditions.

### 8.5 Green Tea and Exercise: Antioxidant Modulation With Limited Additive Cognitive Effects

Green tea, rich in catechins such as epigallocatechin gallate, is widely recognized for its antioxidant and anti‐inflammatory properties. When combined with physical exercise, green tea has been proposed to offer enhanced neuroprotection, particularly through redox modulation and potential support of synaptic function. However, current evidence suggests that while both interventions are beneficial independently, their combined effects on hippocampal health may not always be additive. In an aging rodent model, Flôres et al. (2014) evaluated the effects of regular exercise and green tea supplementation on memory performance and oxidative stress in the hippocampus. Exercise alone improved cognitive function and reduced oxidative markers, whereas green tea further enhanced antioxidant enzyme activity. However, no significant additive benefit was observed on spatial memory when the two interventions were combined. These results suggest that while green tea strengthens the brain's antioxidant defense, it may not substantially augment exercise‐induced cognitive improvements under certain conditions (Flôres et al. 2014).

Complementary findings were reported by Mao and Thanaphonganan (2024) in a study examining the combined effects of green tea extract and structured physical training in healthy college students. Although the study focused primarily on physical performance, participants receiving green tea extract alongside exercise showed the greatest improvements in anaerobic power and fatigue resistance, particularly among female participants. Although not directly targeting the hippocampus, these results suggest systemic physiological benefits that could potentially translate to central nervous system support with long‐term intervention (Mao and Thanaphonganan 2024). Thus, the combination of green tea and exercise appears to exert complementary effects on antioxidant capacity and general physiological resilience. However, current data, particularly from aging animal models, indicate that green tea does not consistently enhance exercise‐induced cognitive gains. Further research is needed to explore whether dose, timing, or exercise intensity may influence the interaction between these two interventions in hippocampal function.

### 8.6 Soy‐Based Nutrition and Exercise: Dietary Fat Balance and Synaptic Support Across the Lifespan

Soy‐based foods, rich in bioactive peptides, isoflavones, and unsaturated fatty acids, have been investigated for their potential to support brain health, particularly in relation to synaptic plasticity and cognitive performance. When combined with physical activity, soy‐derived nutrition may help regulate neurotrophic signaling, neurotransmission, and hippocampal function—especially under conditions of early cognitive decline or metabolic imbalance. In a study by Cheng et al. (2018), young spontaneously hypertensive rats fed a low‐soybean‐oil diet displayed impaired spatial learning, reduced hippocampal BDNF expression, and decreased NMDA receptor activity. Introduction of a swimming regimen reversed many of these deficits, restoring hippocampal signaling and improving performance on memory tasks. These findings suggest that exercise can compensate for suboptimal soy intake by enhancing neuroplastic mechanisms that are otherwise disrupted by dietary imbalances (Cheng et al. 2018). Teixeira et al. (2011) further explored the effects of different dietary fats, including soybean oil, on cognitive performance and anxiety‐related behaviors in rats undergoing swimming exercise. Animals consuming soybean oil in conjunction with regular exercise performed better in spatial memory tasks and exhibited reduced anxiety‐like behavior compared to those fed saturated or trans fats. Exercise also appeared to mitigate the harmful impact of hydrogenated fats on hippocampal Na^+^/K^+^‐ATPase activity, a key enzyme involved in synaptic function and membrane integrity (Teixeira et al. 2011).

In human studies, Imaoka et al. (2022) investigated the effects of soy peptide supplementation combined with structured exercise in older adults. Over 3 months, participants who received both interventions demonstrated small but statistically significant improvements in memory performance, as well as high adherence and tolerability (Imaoka et al. 2022). An earlier trial by the same group showed greater gains in executive function, calculation ability, and physical strength in older individuals undergoing multicomponent training with soy peptide supplementation compared to exercise alone. These findings point to a dual benefit for both brain and physical health in aging populations (Imaoka et al. 2019). These results support the potential of combining soy‐based nutrition with regular physical activity to enhance hippocampal function. Through mechanisms involving neurotrophic factor regulation, synaptic enzyme activity, and dietary fat modulation, this integrative approach may offer a practical, non‐pharmacological strategy for supporting memory, cognition, and emotional resilience across the lifespan.

### 8.7 Broader Polyphenol‐Rich Diets and Exercise: Neuroplasticity and Inflammation Control in Human and Animal Models

Beyond individual compounds, diets rich in polyphenols such as those found in berries, grapes, teas, and seeds, are increasingly recognized for their ability to enhance brain health, particularly when paired with regular physical activity. These interventions appear to converge on key neurobiological pathways, including BDNF signaling, inflammation regulation, and oxidative stress control, with the hippocampus emerging as a central site of benefit. Cheatham et al. (2022) reviewed the role of flavonoids in brain function, emphasizing their conversion into active metabolites by the gut microbiota. These metabolites not only enhance microbial diversity but may also modulate neuroinflammation and support cognitive processes such as memory, particularly in older adults. This points to a possible gut‐brain mechanism through which polyphenols interact with exercise‐induced neuroplasticity (Cheatham et al. 2022). Trevizol et al. (2018) examined the effects of grape juice supplementation, a polyphenol‐rich beverage, in older women undergoing physical training. Grape juice alone increased serum BDNF, but cognitive improvements, particularly in memory performance, were only observed when supplementation was combined with exercise. These findings suggest that physical activity may be necessary to translate molecular changes into functional cognitive outcomes (Trevizol et al. 2018). Shalan et al. (2021, 2020) evaluated various plant‐based supplements, such as black mulberry, sunflower, and pumpkin seeds, on cognitive function in young adults. Participants who received supplementation with or without exercise showed improvements in attention and memory, but the combination group exhibited greater BDNF upregulation and reduced expression of glucocorticoid receptors, suggesting enhanced stress resilience. However, in some domains, supplementation alone yielded comparable outcomes to the combined intervention, indicating that the effects of polyphenols may be context‐specific (Shalan et al. 2021, 2020).

In aging mouse models, Gibbons (2014) found that voluntary running significantly improved hippocampal neurogenesis and learning, whereas supplementation with EGCG or β‐alanine did not enhance these outcomes beyond those achieved by exercise. This suggests that exercise may exert more consistent effects on hippocampal plasticity in older subjects, whereas dietary polyphenols provide complementary, rather than additive, support (Gibbons 2014). These studies highlight the value of integrating polyphenol‐rich dietary patterns with regular physical activity as a holistic strategy for promoting hippocampal health and cognitive longevity. Although the degree of synergy varies by compound, age, and intervention context, both lifestyle factors influence overlapping pathways, most notably BDNF signaling, oxidative defense, and inflammatory modulation. Future research should aim to clarify optimal combinations and intervention parameters to maximize their neuroprotective potential.

## 9 Limitations of Current Evidence

Although the current body of literature strongly supports the role of physical exercise and polyphenol supplementation in promoting hippocampal neurogenesis and cognitive resilience, several limitations must be acknowledged. First, a significant proportion of the evidence arises from preclinical animal models, which may not fully replicate the complexity of human aging and disease processes. Differences in metabolism, brain structure, and lifespan between rodents and humans limit direct translatability of findings. Second, many studies employ isolated polyphenolic compounds at pharmacological doses that may not reflect achievable levels through dietary intake alone. The bioavailability, metabolism, and tissue distribution of polyphenols in humans are influenced by numerous variables, including gut microbiota composition, making standardized dosing and comparison across studies challenging. Additionally, heterogeneity in exercise protocols—ranging in intensity, duration, and type—complicates efforts to identify optimal regimens for cognitive enhancement. Few studies directly compare modalities such as aerobic exercise, resistance training, and HIIT under standardized conditions. Furthermore, sex differences and age‐specific responses to interventions are frequently underexplored, despite growing evidence that these factors significantly influence neuroplastic outcomes. Human trials remain limited in number, sample size, and duration, with many focusing on surrogate biomarkers rather than long‐term cognitive endpoints. Finally, mechanistic studies in humans are scarce, and few investigations directly assess molecular markers of neurogenesis, such as BDNF, in conjunction with imaging or behavioral data.

## 10 Future Research Directions and Clinical Implications

Future research should prioritize well‐designed, longitudinal human studies that combine neuropsychological assessments with neuroimaging and biomarker analysis to better capture the real‐world impact of exercise and polyphenols on hippocampal function. There is also a need to refine our understanding of dose–response relationships—both for exercise (e.g., intensity and frequency) and for polyphenol intake (e.g., specific compounds, combinations, and delivery systems). Investigating the interaction between genetic background, sex, microbiota composition, and intervention efficacy may offer personalized approaches to brain health promotion. Further elucidation of molecular mechanisms is warranted, especially in clinical populations with neurodegenerative or mood disorders. This includes a deeper exploration of pathways such as autophagy, sirtuin activity, mitochondrial biogenesis, and epigenetic modulation. The synergistic potential of combining physical activity with polyphenol‐rich diets should be tested in diverse populations, with attention to lifestyle factors that may enhance or hinder outcomes. Importantly, future work should address the feasibility, adherence, and cost‐effectiveness of such interventions to facilitate their integration into public health strategies for healthy aging and cognitive preservation. Collectively, expanding the evidence base in these areas will be essential to translate promising experimental findings into practical, scalable, and individualized interventions that improve cognitive health across the lifespan.

## 11 Conclusions

Accumulating evidence from experimental and clinical research indicates that physical exercise and dietary polyphenols influence hippocampal plasticity through partially overlapping, yet mechanistically distinct, molecular pathways that regulate adult neurogenesis, synaptic integrity, and cognitive performance during aging. Among these mechanisms, exercise‐induced upregulation of BDNF represents the most consistently supported pathway, with robust evidence demonstrating its role in neural stem cell proliferation, neuronal differentiation, dendritic maturation, and activity‐dependent synaptic remodeling within the dentate gyrus. Activation of TrkB receptors by BDNF initiates downstream signaling cascades, including PI3K/Akt and MAPK/ERK, which collectively promote neuronal survival, enhance synaptic efficacy, and counteract age‐related reductions in hippocampal plasticity. Beyond BDNF signaling, physical exercise modulates additional pathways that contribute to the maintenance of the neurogenic niche. Wnt/β‐catenin signaling, through inhibition of GSK‐3β and stabilization of β‐catenin, supports progenitor cell proliferation and neuronal lineage commitment, whereas Notch signaling preserves the neural stem cell pool and regulates fate specification. Although these pathways are increasingly implicated in exercise‐induced neurogenesis, their characterization remains largely preclinical, and their direct contribution to human hippocampal neurogenesis requires further validation. Exercise also exerts indirect neurogenic effects by improving cerebral perfusion via VEGF‐mediated angiogenesis, regulating glucocorticoid receptor signaling to limit stress‐induced neurogenic suppression, and activating autophagy‐related pathways that sustain cellular homeostasis in aging neural progenitors. Dietary polyphenols contribute complementary neuroprotective actions by targeting oxidative stress, neuroinflammation, and mitochondrial dysfunction, key processes that constrain neurogenesis in the aging brain. Polyphenols such as resveratrol, curcumin, quercetin, and catechins modulate redox‐sensitive transcription factors, including Nrf2 and NF‐κB, thereby reducing pro‐inflammatory cytokine signaling and preserving the neurogenic microenvironment. At the metabolic level, several polyphenols activate SIRT1‐ and AMPK‐dependent pathways, enhancing mitochondrial biogenesis, improving NAD^+^ availability, and stabilizing neuronal energy metabolism, processes that are critical for sustaining neural stem cell function and synaptic transmission. Certain flavonoids further interact with neurotrophic signaling by enhancing BDNF expression or mimicking TrkB activation, providing a mechanistic link between nutritional bioactives and exercise‐responsive pathways.

Evidence from combined intervention studies suggests that exercise and polyphenols may act additively or synergistically, particularly under conditions of metabolic stress, neurotoxicity, chronic inflammation, or neurodegenerative pathology. In these contexts, exercise primarily drives activity‐dependent neuroplastic signaling, whereas polyphenols attenuate molecular constraints on neurogenesis, such as oxidative damage, endoplasmic reticulum stress, and apoptotic signaling. However, it is important to emphasize that the majority of synergistic effects have been demonstrated in animal models, and human studies to date predominantly report improvements in cognitive performance, mood, and circulating neurotrophic markers rather than direct evidence of enhanced hippocampal neurogenesis. Translational interpretation of these findings therefore warrants caution. Variability in polyphenol bioavailability, differences in exercise modality and intensity, and the current inability to directly quantify neurogenesis in the living human brain limit definitive conclusions regarding mechanism‐specific efficacy. Nonetheless, the convergence of animal and human data on shared, lifestyle‐responsive pathways, notably BDNF signaling, inflammatory modulation, and mitochondrial regulation, supports the biological plausibility of integrating structured physical activity with polyphenol‐rich dietary patterns as adjunct strategies for maintaining cognitive health during aging. Future research should prioritize long‐term, well‐controlled human trials that integrate nutritional intervention, exercise prescription, neuroimaging, and molecular biomarkers, including circulating neurotrophins, inflammatory mediators, and metabolic signatures. Such integrative approaches will be essential to delineate dose–response relationships, identify responder subgroups, and clarify the relative contribution of established versus emerging mechanisms. From a nutritional science perspective, these findings highlight the potential of diet–lifestyle interactions to modulate brain aging through defined molecular pathways, offering a rational foundation for evidence‐based, non‐pharmacological strategies aimed at preserving hippocampal function and cognitive resilience across the lifespan.

## Author Contributions

**Zhenyi Zhao:** conceptualization, investigation, writing – original draft, writing – review editing, visualization, methodology, validation, supervision, data curation. **Sima‐sadat Sabihi:** data curation, supervision, conceptualization, investigation, writing – original draft, writing – review editing, visualization, validation, methodology.

## Funding

The authors have nothing to report.

## Conflicts of Interest

The authors declare no conflicts of interest.

## Data Availability

Data sharing not applicable to this article as no datasets were generated or analyzed during the current study.

## References

[fsn371613-bib-0001] Abbott, N. J. , A. A. Patabendige , D. E. Dolman , S. R. Yusof , and D. J. Begley . 2010. “Structure and Function of the Blood–Brain Barrier.” Neurobiology of Disease 37, no. 1: 13–25. 19664713 10.1016/j.nbd.2009.07.030

[fsn371613-bib-0002] Abdallah, H. M. , R. M. El‐Gohary , H. Khattab , et al. 2024. “Potential Synergestic Effect of Quercetin and Exercise on Depressive Like Behavior in Male Albino Rats: Targeting Oxidative Stress, Inflammation, Neural Apoptosis and Mitophagy.” Bulletin of the Egyptian Society for Physiological Sciences 44, no. 2: 54–68. 10.21608/besps.2023.243730.1157.

[fsn371613-bib-0003] Aguilar‐Garcia, I. G. , J. Alpirez , R. Castañeda‐Arellano , et al. 2024. “Resveratrol and Exercise Produce Recovered Ankle and Metatarsus Joint Movements After Penetrating Lesion in Hippocampus in Male Rats.” Brain Sciences 14, no. 10: 980. 10.3390/brainsci14100980. 39451994 PMC11506448

[fsn371613-bib-0004] Ahmadi, E. , A. Pourmotabbed , N. Aghaz , et al. 2024. “Curcumin and Exercise Prevent Depression via Alleviating Hippocampus Injury and Improve Depressive‐Like Behaviors in Chronically Stressed Depression Rats.” Research in Pharmaceutical Sciences 19, no. 5: 509–519. 10.4103/rps.rps_94_23. 39691296 PMC11648346

[fsn371613-bib-0005] Akbari‐Fakhrabadi, M. , M. Najafi , S. Mortazavian , et al. 2021. “Saffron (*Crocus sativus* L.), Combined With Endurance Exercise, Synergistically Enhances BDNF, Serotonin, and NT‐3 in Wistar Rats.” Reports of Biochemistry & Molecular Biology 9, no. 4: 426–434. 10.52547/rbmb.9.4.426. 33969136 PMC8068454

[fsn371613-bib-0006] Alonso, M. , A.‐C. Petit , and P.‐M. Lledo . 2024. “The Impact of Adult Neurogenesis on Affective Functions: Of Mice and Men.” Molecular Psychiatry 29, no. 8: 2527–2542. 10.1038/s41380-024-02504-w. 38499657 PMC11412911

[fsn371613-bib-0007] Amaral Dias, K. S. , f. eacute , J. A. Ramos , B. M. Gomes , A. A. Santos , and L. C. zio . 2019. “Effect of Curcumin and Physical Training on the Brain and Motor Performance of Rats With Cerebral Ischemia.” Neuroscience International 10, no. 1: 1–7. 10.3844/amjnsp.2019.1.7.

[fsn371613-bib-0008] Amirazodi, M. , A. Mehrabi , M. A. Rajizadeh , et al. 2022. “The Effects of Combined Resveratrol and High Intensity Interval Training on the Hippocampus in Aged Male Rats: An Investigation Into Some Signaling Pathways Related to Mitochondria.” Iranian Journal of Basic Medical Sciences 25, no. 2: 254–262. 10.22038/ijbms.2022.57780.12853. 35655601 PMC9124540

[fsn371613-bib-0009] Anand, K. S. , and V. Dhikav . 2012. “Hippocampus in Health and Disease: An Overview.” Annals of Indian Academy of Neurology 15, no. 4: 239–246. 10.4103/0972-2327.104323. 23349586 PMC3548359

[fsn371613-bib-0010] Angelino, D. , J. Godos , F. Ghelfi , et al. 2019. “Fruit and Vegetable Consumption and Health Outcomes: An Umbrella Review of Observational Studies.” International Journal of Food Sciences and Nutrition 70, no. 6: 652–667. 30764679 10.1080/09637486.2019.1571021

[fsn371613-bib-0011] Arrabal‐Gómez, C. , P. Serrano‐Castro , J. A. Sánchez‐Pérez , et al. 2024. “Potentiation of Antidepressant Effects: NPY1R Agonist and Ketamine Synergy Enhances TrkB Signaling and Neurogenesis in the Ventral Hippocampus.” Expert Opinion on Therapeutic Targets 28, no. 4: 309–322. 10.1080/14728222.2024.2342524. 38626283

[fsn371613-bib-0012] Azarian, F. , S. Farsi , S. A. Hosseini , and M. A. Azarbayjani . 2020. “Effect of Endurance Training With Saffron Consumption on PGC1‐α Gene Expression in Hippocampus Tissue of Rats With Alzheimer's Disease.” Annals of Military and Health Sciences Research 18, no. 1: e99131.

[fsn371613-bib-0013] Badji, A. , J. Youwakim , A. Cooper , E. Westman , and A. Marseglia . 2023. “Vascular Cognitive Impairment—Past, Present, and Future Challenges.” Ageing Research Reviews 90: 102042. 10.1016/j.arr.2023.102042. 37634888

[fsn371613-bib-0014] Barone, D. A. 2024. “Trauma‐Associated Sleep Disorder.” Sleep Medicine Clinics 19, no. 1: 93–99. 10.1016/j.jsmc.2023.10.005. 38368073

[fsn371613-bib-0015] Bazyar Halimehjani, F. , and R. Shabani . 2023. “Aerobic Exercises Along With the Consumption of Saffron Extract on Spatial Memory and the Amount of Tau Accumulation in the Hippocampal Tissue of Male Alzheimer's Rats.” Metabolism and Exercise 13, no. 1: 17–33.

[fsn371613-bib-0016] Becker, S. , and J. M. Wojtowicz . 2007. “A Model of Hippocampal Neurogenesis in Memory and Mood Disorders.” Trends in Cognitive Sciences 11, no. 2: 70–76. 10.1016/j.tics.2006.10.013. 17174137

[fsn371613-bib-0017] Bhattacharjee, D. , V. Chaudhary , K. Khate , N. K. Devi , N. Babu , and K. N. Saraswathy . 2023. “Incidence and Risk Factors of Cognitive Impairment: A 6‐Year Follow‐Up Study From North India.” Clinical Epidemiology and Global Health 24: 101422. 10.1016/j.cegh.2023.101422.

[fsn371613-bib-0018] Boldrini, M. , C. A. Fulmore , A. N. Tartt , et al. 2018. “Human Hippocampal Neurogenesis Persists Throughout Aging.” Cell Stem Cell 22, no. 4: 589–599.e585. 10.1016/j.stem.2018.03.015. 29625071 PMC5957089

[fsn371613-bib-0019] Bond, A. M. , G. L. Ming , and H. Song . 2015. “Adult Mammalian Neural Stem Cells and Neurogenesis: Five Decades Later.” Cell Stem Cell 17, no. 4: 385–395. 10.1016/j.stem.2015.09.003. 26431181 PMC4683085

[fsn371613-bib-0020] Broderick, T. L. , S. Rasool , R. Li , et al. 2020. “Neuroprotective Effects of Chronic Resveratrol Treatment and Exercise Training in the 3xTg‐AD Mouse Model of Alzheimer's Disease.” International Journal of Molecular Sciences 21, no. 19: 7337. 10.3390/ijms21197337. 33020412 PMC7582460

[fsn371613-bib-0021] Carpentier, P. A. , and T. D. Palmer . 2009. “Immune Influence on Adult Neural Stem Cell Regulation and Function.” Neuron 64, no. 1: 79–92. 10.1016/j.neuron.2009.08.038. 19840551 PMC2789107

[fsn371613-bib-0022] Casadesus, G. , B. Shukitt‐Hale , H. M. Stellwagen , et al. 2004. “Modulation of Hippocampal Plasticity and Cognitive Behavior by Short‐Term Blueberry Supplementation in Aged Rats.” Nutritional Neuroscience 7, no. 5–6: 309–316. 15682927 10.1080/10284150400020482

[fsn371613-bib-0023] Caspersen, C. J. , K. E. Powell , and G. M. Christenson . 1985. “Physical Activity, Exercise, and Physical Fitness: Definitions and Distinctions for Health‐Related Research.” Public Health Reports 100, no. 2: 126–131. 3920711 PMC1424733

[fsn371613-bib-0024] Chang, Y. T. , Y. C. Chen , C. W. Wu , et al. 2008. “Glucocorticoid Signaling and Exercise‐Induced Downregulation of the Mineralocorticoid Receptor in the Induction of Adult Mouse Dentate Neurogenesis by Treadmill Running.” Psychoneuroendocrinology 33, no. 9: 1173–1182. 10.1016/j.psyneuen.2008.05.014. 18760539

[fsn371613-bib-0025] Cheatham, C. L. , D. C. Nieman , A. P. Neilson , and M. A. Lila . 2022. “Enhancing the Cognitive Effects of Flavonoids With Physical Activity: Is There a Case for the Gut Microbiome?” Frontiers in Neuroscience 16: 833202. 35273477 10.3389/fnins.2022.833202 PMC8902155

[fsn371613-bib-0026] Chen, D. , Y. Guo , M. Zhang , X. Liu , B. Zhang , and X. Kou . 2025. “Exercise Alleviates Cognitive Decline of Natural Aging Rats by Upregulating Notch‐Mediated Autophagy Signaling.” Brain Research 1850: 149398. 10.1016/j.brainres.2024.149398. 39667553

[fsn371613-bib-0027] Chen, X. , C. Guo , and J. Kong . 2012. “Oxidative Stress in Neurodegenerative Diseases.” Neural Regeneration Research 7, no. 5: 376–385. 25774178 10.3969/j.issn.1673-5374.2012.05.009 PMC4350122

[fsn371613-bib-0028] Cheng, M. , J. Cong , Y. Wu , et al. 2018. “Chronic Swimming Exercise Ameliorates Low‐Soybean‐Oil Diet‐Induced Spatial Memory Impairment by Enhancing BDNF‐Mediated Synaptic Potentiation in Developing Spontaneously Hypertensive Rats.” Neurochemical Research 43, no. 5: 1047–1057. 10.1007/s11064-018-2515-x. 29574667

[fsn371613-bib-0029] Cho, J. A. , S. H. Park , J. Cho , J. O. Kim , J. H. Yoon , and E. Park . 2020. “Exercise and Curcumin in Combination Improves Cognitive Function and Attenuates ER Stress in Diabetic Rats.” Nutrients 12, no. 5: 1309. 10.3390/nu12051309. 32375323 PMC7284733

[fsn371613-bib-0030] Cho, J. W. , S. Y. Jung , D. Y. Kim , et al. 2018. “PI3K‐Akt‐Wnt Pathway Is Implicated in Exercise‐Induced Improvement of Short‐Term Memory in Cerebral Palsy Rats.” International Neurourology Journal 22, no. Suppl 3: S156–S164. 10.5213/inj.1836224.112. 30396265 PMC6234731

[fsn371613-bib-0031] Chung, S. , H. Yao , S. Caito , J.‐w. Hwang , G. Arunachalam , and I. Rahman . 2010. “Regulation of SIRT1 in Cellular Functions: Role of Polyphenols.” Archives of Biochemistry and Biophysics 501, no. 1: 79–90. 20450879 10.1016/j.abb.2010.05.003 PMC2930135

[fsn371613-bib-0032] Cicero, A. F. , F. Fogacci , and M. Banach . 2018. “Botanicals and Phytochemicals Active on Cognitive Decline: The Clinical Evidence.” Pharmacological Research 130: 204–212. 29289576 10.1016/j.phrs.2017.12.029

[fsn371613-bib-0033] Cole, J. H. , R. E. Marioni , S. E. Harris , and I. J. Deary . 2019. “Brain Age and Other Bodily ‘ages’: Implications for Neuropsychiatry.” Molecular Psychiatry 24, no. 2: 266–281. 10.1038/s41380-018-0098-1. 29892055 PMC6344374

[fsn371613-bib-0034] Culig, L. , X. Chu , and V. A. Bohr . 2022. “Neurogenesis in Aging and Age‐Related Neurodegenerative Diseases.” Ageing Research Reviews 78: 101636. 10.1016/j.arr.2022.101636. 35490966 PMC9168971

[fsn371613-bib-0035] Dastgerdi, H. H. , M. Radahmadi , P. Reisi , and A. H. Dastgerdi . 2018. “Effect of Crocin, Exercise, and Crocin‐Accompanied Exercise on Learning and Memory in Rats Under Chronic Unpredictable Stress.” Advanced Biomedical Research 7, no. 1: 137. 30464937 10.4103/abr.abr_153_18 PMC6206744

[fsn371613-bib-0036] Dias, G. P. , N. Cavegn , A. Nix , et al. 2012. “The Role of Dietary Polyphenols on Adult Hippocampal Neurogenesis: Molecular Mechanisms and Behavioural Effects on Depression and Anxiety.” Oxidative Medicine and Cellular Longevity 2012, no. 1: 541971. 22829957 10.1155/2012/541971 PMC3395274

[fsn371613-bib-0037] Du Preez, A. , S. Lefèvre‐Arbogast , R. González‐Domínguez , et al. 2024. “Association of Dietary and Nutritional Factors With Cognitive Decline, Dementia, and Depressive Symptomatology in Older Individuals According to a Neurogenesis‐Centred Biological Susceptibility to Brain Ageing.” Age and Ageing 53, no. Suppl 2: ii47–ii59. 10.1093/ageing/afae042. 38745492 PMC11094407

[fsn371613-bib-0038] Elhampour, L. , M. A. Azarbayjani , M. Nasehi , and M. Peeri . 2019. “Concurrent Effects of Exercise and Curcumin on Spatial Learning and Memory in Sensitized Male Mice Following Morphine Administration.” Galen Medical Journal 8: e1072. 10.31661/gmj.v8i0.1072. 34466459 PMC8343884

[fsn371613-bib-0039] Eriksson, P. S. , E. Perfilieva , T. Björk‐Eriksson , et al. 1998. “Neurogenesis in the Adult Human Hippocampus.” Nature Medicine 4, no. 11: 1313–1317. 10.1038/3305. 9809557

[fsn371613-bib-0040] Eshkoor, S. A. , T. A. Hamid , C. Y. Mun , and C. K. Ng . 2015. “Mild Cognitive Impairment and Its Management in Older People.” Clinical Interventions in Aging 10: 687–693. 10.2147/cia.s73922. 25914527 PMC4401355

[fsn371613-bib-0041] Fan, D. , J. Li , B. Zheng , L. Hua , and Z. Zuo . 2016. “Enriched Environment Attenuates Surgery‐Induced Impairment of Learning, Memory, and Neurogenesis Possibly by Preserving BDNF Expression.” Molecular Neurobiology 53, no. 1: 344–354. 10.1007/s12035-014-9013-1. 25432890

[fsn371613-bib-0042] Flanagan, E. , M. Müller , M. Hornberger , and D. Vauzour . 2018. “Impact of Flavonoids on Cellular and Molecular Mechanisms Underlying Age‐Related Cognitive Decline and Neurodegeneration.” Current Nutrition Reports 7: 49–57. 29892788 10.1007/s13668-018-0226-1 PMC5960493

[fsn371613-bib-0043] Flôres, M. , A. Martins , H. Schimidt , et al. 2014. “Effects of Green Tea and Physical Exercise on Memory Impairments Associated With Aging.” Neurochemistry International 78: 53–60. 10.1016/j.neuint.2014.08.008. 25195719

[fsn371613-bib-0044] Flor‐García, M. , J. Terreros‐Roncal , E. P. Moreno‐Jiménez , J. Ávila , A. Rábano , and M. Llorens‐Martín . 2020. “Unraveling Human Adult Hippocampal Neurogenesis.” Nature Protocols 15, no. 2: 668–693. 10.1038/s41596-019-0267-y. 31915385

[fsn371613-bib-0045] Frota, N. A. F. , E. R. Barbosa , C. S. Porto , et al. 2016. “Which Factors Are Associated With Global Cognitive Impairment in Wilson's Disease?” Dementia & Neuropsychologia 10, no. 4: 320–326. 10.1590/s1980-5764-2016dn1004011. 29213476 PMC5619272

[fsn371613-bib-0046] Gao, Y. , M. Syed , and X. Zhao . 2023. “Mechanisms Underlying the Effect of Voluntary Running on Adult Hippocampal Neurogenesis.” Hippocampus 33, no. 4: 373–390. 10.1002/hipo.23520. 36892196 PMC10566571

[fsn371613-bib-0047] Gibbons, T. 2014. Enhancing Learning and Memory in the Aged: Interactions Between Dietary Supplementation and Exercise. University of Illinois at Urbana‐Champaign.

[fsn371613-bib-0048] Glass, C. K. , K. Saijo , B. Winner , M. C. Marchetto , and F. H. Gage . 2010. “Mechanisms Underlying Inflammation in Neurodegeneration.” Cell 140, no. 6: 918–934. 20303880 10.1016/j.cell.2010.02.016 PMC2873093

[fsn371613-bib-0049] Grabska‐Kobyłecka, I. , P. Szpakowski , A. Król , et al. 2023. “Polyphenols and Their Impact on the Prevention of Neurodegenerative Diseases and Development.” Nutrients 15, no. 15: 3454. 37571391 10.3390/nu15153454 PMC10420887

[fsn371613-bib-0050] Granzotto, A. , and P. Zatta . 2014. “Resveratrol and Alzheimer's Disease: Message in a Bottle on Red Wine and Cognition.” Frontiers in Aging Neuroscience 6: 95. 24860502 10.3389/fnagi.2014.00095 PMC4030174

[fsn371613-bib-0051] Guo, Y. J. , Z. J. Zhang , S. H. Wang , Y. X. Sui , and Y. Sun . 2009. “Notch1 Signaling, Hippocampal Neurogenesis and Behavioral Responses to Chronic Unpredicted Mild Stress in Adult Ischemic Rats.” Progress in Neuro‐Psychopharmacology & Biological Psychiatry 33, no. 4: 688–694. 10.1016/j.pnpbp.2009.03.022. 19336246

[fsn371613-bib-0052] Habibi, S. , A. Abdi , and S. Saeid Fazelifar . 2023. “The Effect of Aerobic Training and Resveratrol on Ferroptosis in a Rat Model of Alzheimer's Disease. [اثر تمرین هوازی و رزوراترول بر فروپتوز در مدل موش‌های صحرایی مبتلا به آلزایمر].” Neuroscience‐Journal‐of‐Shefaye‐Khatam 11, no. 4: 1. 10.61186/shefa.11.4.1.

[fsn371613-bib-0053] Habibian, M. , S. J. Moosavi , and P. Farzanegi . 2016. “Regular Exercise Combined With Curcumin Supplementation: Protective Effects Against Lead‐Induced Cerebellar Oxidative Damage in an Animal Model.” Neurophysiology 48, no. 1: 17–22. 10.1007/s11062-016-9564-z.

[fsn371613-bib-0054] Hagg, T. 2009. “From Neurotransmitters to Neurotrophic Factors to Neurogenesis.” Neuroscientist 15, no. 1: 20–27. 10.1177/1073858408324789. 19218228 PMC2722065

[fsn371613-bib-0055] Hagihara, H. , T. Murano , K. Ohira , M. Miwa , K. Nakamura , and T. Miyakawa . 2019. “Expression of Progenitor Cell/Immature Neuron Markers Does Not Present Definitive Evidence for Adult Neurogenesis.” Molecular Brain 12, no. 1: 108. 10.1186/s13041-019-0522-8. 31823803 PMC6902531

[fsn371613-bib-0056] Hosseinlou, A. , R. Pouzesh Jadidi , J. Bashiri , M. A. R. Nourazar , and K. Azali‐ Alamdari . 2020. “Effects of High Intensity Interval Training and Curcumin Supplementation on Hippocampal Total Antioxidant Capacity, GRP78 and Caspase 3 Level in Male Rats Exposed to Arsenic.” Journal of Applied Exercise Physiology 16, no. 32: 15–29. 10.22080/jaep.2020.18555.1943.

[fsn371613-bib-0057] Hosseinzadeh, S. , V. D. Roshan , and S. Mahjoub . 2013. “Continuous Exercise Training and Curcumin Attenuate Changes in Brain‐Derived Neurotrophic Factor and Oxidative Stress Induced by Lead Acetate in the Hippocampus of Male Rats.” Pharmaceutical Biology 51, no. 2: 240–245. 10.3109/13880209.2012.717230. 23134146

[fsn371613-bib-0058] Hu, Z. , J. Ma , H. Yue , et al. 2022. “Involvement of LIN28A in Wnt‐Dependent Regulation of Hippocampal Neurogenesis in the Aging Brain.” Stem Cell Reports 17, no. 7: 1666–1682. 10.1016/j.stemcr.2022.05.016. 35750042 PMC9287676

[fsn371613-bib-0059] Huang, Y. Y. , S. D. Chen , X. Y. Leng , et al. 2022. “Post‐Stroke Cognitive Impairment: Epidemiology, Risk Factors, and Management.” Journal of Alzheimer's Disease 86, no. 3: 983–999. 10.3233/jad-215644. 35147548

[fsn371613-bib-0060] Huhn, S. , F. Beyer , R. Zhang , et al. 2018. “Effects of Resveratrol on Memory Performance, Hippocampus Connectivity and Microstructure in Older Adults—A Randomized Controlled Trial.” NeuroImage 174: 177–190. 10.1016/j.neuroimage.2018.03.023. 29548848

[fsn371613-bib-0062] Imaoka, M. , H. Nakao , M. Nakamura , et al. 2019. “Effect of Multicomponent Exercise and Nutrition Support on the Cognitive Function of Older Adults: A Randomized Controlled Trial.” Clinical Interventions in Aging 14: 2145–2153. 31849458 10.2147/CIA.S229034 PMC6912002

[fsn371613-bib-0061] Imaoka, M. , H. Nakao , M. Nakamura , et al. 2022. “Improvement of Memory Function via a Combination of Exercise and Soy Peptide Supplementation in Community‐Dwelling Older Adults: A Randomized Controlled Trial.” Contemporary Clinical Trials Communications 30: 100998. 10.1016/j.conctc.2022.100998. 36124312 PMC9482121

[fsn371613-bib-0063] Inoue, K. , M. Okamoto , J. Shibato , et al. 2015. “Long‐Term Mild, Rather Than Intense, Exercise Enhances Adult Hippocampal Neurogenesis and Greatly Changes the Transcriptomic Profile of the Hippocampus.” PLoS One 10, no. 6: e0128720. 10.1371/journal.pone.0128720. 26061528 PMC4464753

[fsn371613-bib-0064] Ito, H. , X.‐L. Sun , M. Watanabe , M. Okamoto , and T. Hatano . 2008. “Chlorogenic Acid and Its Metabolite m‐Coumaric Acid Evoke Neurite Outgrowth in Hippocampal Neuronal Cells.” Bioscience, Biotechnology, and Biochemistry 72, no. 3: 885–888. 18323641 10.1271/bbb.70670

[fsn371613-bib-0065] Jachim, S. K. , A. E. Sakamoto , X. Zhang , V. M. Pearsall , M. J. Schafer , and N. K. LeBrasseur . 2020. “Harnessing the Effects of Endurance Exercise to Optimize Cognitive Health: Fundamental Insights From Dr. Mark P. Mattson.” Ageing Research Reviews 64: 101147. 10.1016/j.arr.2020.101147. 32814127 PMC7710559

[fsn371613-bib-0066] Jang, Y. 2020. “Endurance Exercise‐Induced Expression of Autophagy‐Related Protein Coincides With Anabolic Expression and Neurogenesis in the Hippocampus of the Mouse Brain.” Neuroreport 31, no. 6: 442–449. 10.1097/wnr.0000000000001431. 32168100

[fsn371613-bib-0067] Ji, E. S. , Y. M. Kim , Y. J. Ko , and S. S. Baek . 2020. “Treadmill Exercise in Obese Maternal Rats During Pregnancy Improves Short‐Term Memory Through Neurogenesis in the Hippocampus of Rat Pups.” Journal of Exercise Rehabilitation 16, no. 5: 392–397. 10.12965/jer.2040618.309. 33178640 PMC7609846

[fsn371613-bib-0068] Jiang, B. , Z. Xiong , J. Yang , et al. 2012. “Antidepressant‐Like Effects of Ginsenoside Rg1 Are due to Activation of the BDNF Signalling Pathway and Neurogenesis in the Hippocampus.” British Journal of Pharmacology 166, no. 6: 1872–1887. 10.1111/j.1476-5381.2012.01902.x. 22335772 PMC3402811

[fsn371613-bib-0069] Johansson, M. M. , J. Marcusson , and E. Wressle . 2015. “Cognitive Impairment and Its Consequences in Everyday Life: Experiences of People With Mild Cognitive Impairment or Mild Dementia and Their Relatives.” International Psychogeriatrics 27, no. 6: 949–958. 10.1017/S1041610215000058. 25644289

[fsn371613-bib-0070] Karuppagounder, S. , S. Madathil , M. Pandey , R. Haobam , U. Rajamma , and K. Mohanakumar . 2013. “Quercetin Up‐Regulates Mitochondrial Complex‐I Activity to Protect Against Programmed Cell Death in Rotenone Model of Parkinson's Disease in Rats.” Neuroscience 236: 136–148. 23357119 10.1016/j.neuroscience.2013.01.032

[fsn371613-bib-0071] Kean, R. J. , D. J. Lamport , G. F. Dodd , et al. 2015. “Chronic Consumption of Flavanone‐Rich Orange Juice Is Associated With Cognitive Benefits: An 8‐wk, Randomized, Double‐Blind, Placebo‐Controlled Trial in Healthy Older Adults.” American Journal of Clinical Nutrition 101, no. 3: 506–514. 25733635 10.3945/ajcn.114.088518

[fsn371613-bib-0072] Khurana, S. , K. Venkataraman , A. Hollingsworth , M. Piche , and T. Tai . 2013. “Polyphenols: Benefits to the Cardiovascular System in Health and in Aging.” Nutrients 5, no. 10: 3779–3827. 24077237 10.3390/nu5103779 PMC3820045

[fsn371613-bib-0073] Kim, D. Y. , S. Y. Jung , K. Kim , and C. J. Kim . 2016. “Treadmill Exercise Ameliorates Alzheimer Disease‐Associated Memory Loss Through the Wnt Signaling Pathway in the Streptozotocin‐Induced Diabetic Rats.” Journal of Exercise Rehabilitation 12, no. 4: 276–283. 10.12965/jer.1632678.339. 27656623 PMC5031391

[fsn371613-bib-0074] Kiuchi, T. , H. Lee , and T. Mikami . 2012. “Regular Exercise Cures Depression‐Like Behavior via VEGF‐Flk‐1 Signaling in Chronically Stressed Mice.” Neuroscience 207: 208–217. 10.1016/j.neuroscience.2012.01.023. 22306286

[fsn371613-bib-0075] le Tang, X. , X. Wang , G. Fang , et al. 2021. “Resveratrol Ameliorates Sevoflurane‐Induced Cognitive Impairment by Activating the SIRT1/NF‐κB Pathway in Neonatal Mice.” Journal of Nutritional Biochemistry 90: 108579. 33388350 10.1016/j.jnutbio.2020.108579

[fsn371613-bib-0076] Liu, J.‐K. 2022. “Antiaging Agents: Safe Interventions to Slow Aging and Healthy Life Span Extension.” Natural Products and Bioprospecting 12, no. 1: 18. 35534591 10.1007/s13659-022-00339-y PMC9086005

[fsn371613-bib-0077] Liu, S. , Q. Yu , Z. Li , et al. 2020. “Effects of Acute and Chronic Exercises on Executive Function in Children and Adolescents: A Systemic Review and Meta‐Analysis.” Frontiers in Psychology 11: 554915. 33391074 10.3389/fpsyg.2020.554915 PMC7773601

[fsn371613-bib-0078] Longo, F. M. , and S. M. Massa . 2013. “Small‐Molecule Modulation of Neurotrophin Receptors: A Strategy for the Treatment of Neurological Disease.” Nature Reviews Drug Discovery 12, no. 7: 507–525. 23977697 10.1038/nrd4024

[fsn371613-bib-0079] Lucassen, P. J. , C. P. Fitzsimons , E. Salta , and M. Maletic‐Savatic . 2020. “Adult Neurogenesis, Human After All (Again): Classic, Optimized, and Future Approaches.” Behavioural Brain Research 381: 112458. 10.1016/j.bbr.2019.112458. 31899214

[fsn371613-bib-0080] Mandel, S. , T. Amit , L. Reznichenko , O. Weinreb , and M. B. Youdim . 2006. “Green Tea Catechins as Brain‐Permeable, Natural Iron Chelators‐Antioxidants for the Treatment of Neurodegenerative Disorders.” Molecular Nutrition & Food Research 50, no. 2: 229–234. 16470637 10.1002/mnfr.200500156

[fsn371613-bib-0081] Mao, Q. , and N. Thanaphonganan . 2024. “Effect of Green Tea Extract in Conjunction With Exercise Prices of Training on Anaerobic Performances in College Students' Outcomes.” Journal of Ecohumanism 3, no. 8: 7568–7582. 10.62754/joe.v3i8.5385.

[fsn371613-bib-0082] Mattova, S. , P. Simko , N. Urbanska , and T. Kiskova . 2023. “Bioactive Compounds and Their Influence on Postnatal Neurogenesis.” International Journal of Molecular Sciences 24, no. 23: 16614. 38068936 10.3390/ijms242316614 PMC10706651

[fsn371613-bib-0083] Molaei, A. , H. Hatami , G. Dehghan , R. Sadeghian , and N. Khajehnasiri . 2020. “Synergistic Effects of Quercetin and Regular Exercise on the Recovery of Spatial Memory and Reduction of Parameters of Oxidative Stress in Animal Model of Alzheimer's Disease.” EXCLI Journal 19: 596–612. 10.17179/excli2019-2082. 32483406 PMC7257248

[fsn371613-bib-0084] Moosavi, F. , R. Hosseini , L. Saso , and O. Firuzi . 2015. “Modulation of Neurotrophic Signaling Pathways by Polyphenols.” Drug Design, Development and Therapy 10: 23–42. 26730179 10.2147/DDDT.S96936 PMC4694682

[fsn371613-bib-0085] Moriya, J. , R. Chen , J.‐i. Yamakawa , K. Sasaki , Y. Ishigaki , and T. Takahashi . 2011. “Resveratrol Improves Hippocampal Atrophy in Chronic Fatigue Mice by Enhancing Neurogenesis and Inhibiting Apoptosis of Granular Cells.” Biological & Pharmaceutical Bulletin 34, no. 3: 354–359. 10.1248/bpb.34.354. 21372384

[fsn371613-bib-0086] Nokia, M. S. , S. Lensu , J. P. Ahtiainen , et al. 2016. “Physical Exercise Increases Adult Hippocampal Neurogenesis in Male Rats Provided It Is Aerobic and Sustained.” Journal of Physiology 594, no. 7: 1855–1873. 26844666 10.1113/JP271552 PMC4818598

[fsn371613-bib-0087] Noruzi, S. , Z. Meshkati , R. Badami , and R. Nasiri . 2023. “The Effect of High Intensity Interval Training (HIIT) and Dietary Supplement Curcumin on Cognitive Function and the Level of Stress Markers in the Brain of Male Balb/C Mice Exposed to Lead Nitrate.” Motor Behavior 15, no. 52: 69–100. 10.22089/mbj.2023.13675.2056.

[fsn371613-bib-0088] Ogunsuyi, O. B. , P. O. Aro , H. I. Umar , and G. Oboh . 2023. “Administration of Curcumin Plus Treadmill Exercise Modulate Some Neuronal Enzyme Activities, Redox Markers and BDNF mRNA Expression in Pilocarpine‐Induced Epileptic Seizure in Rats.” Neurochemical Journal 17, no. 3: 467–476. 10.1134/S1819712423030145.

[fsn371613-bib-0089] Okamoto, M. , D. Mizuuchi , K. Omura , et al. 2021. “High‐Intensity Intermittent Training Enhances Spatial Memory and Hippocampal Neurogenesis Associated With BDNF Signaling in Rats.” Cerebral Cortex 31, no. 9: 4386–4397. 10.1093/cercor/bhab093. 33982757

[fsn371613-bib-0090] Oomen, C. A. , P. Bekinschtein , B. A. Kent , L. M. Saksida , and T. J. Bussey . 2014. “Adult Hippocampal Neurogenesis and Its Role in Cognition.” Wiley Interdisciplinary Reviews: Cognitive Science 5, no. 5: 573–587. 10.1002/wcs.1304. 26308746 PMC4568304

[fsn371613-bib-0091] Pais, R. , L. Ruano , O. P. Carvalho , and H. Barros . 2020. “Global Cognitive Impairment Prevalence and Incidence in Community Dwelling Older Adults—A Systematic Review.” Geriatrics (Basel) 5, no. 4: 84. 10.3390/geriatrics5040084. 33121002 PMC7709591

[fsn371613-bib-0092] Pérez Palmer, N. , B. Trejo Ortega , and P. Joshi . 2022. “Cognitive Impairment in Older Adults: Epidemiology, Diagnosis, and Treatment.” Psychiatric Clinics of North America 45, no. 4: 639–661. 10.1016/j.psc.2022.07.010. 36396270

[fsn371613-bib-0093] Pervin, M. , K. Unno , T. Ohishi , H. Tanabe , N. Miyoshi , and Y. Nakamura . 2018. “Beneficial Effects of Green Tea Catechins on Neurodegenerative Diseases.” Molecules 23, no. 6: 1297. 29843466 10.3390/molecules23061297 PMC6099654

[fsn371613-bib-0094] Potì, F. , D. Santi , G. Spaggiari , F. Zimetti , and I. Zanotti . 2019. “Polyphenol Health Effects on Cardiovascular and Neurodegenerative Disorders: A Review and Meta‐Analysis.” International Journal of Molecular Sciences 20, no. 2: 351. 30654461 10.3390/ijms20020351 PMC6359281

[fsn371613-bib-0095] Purtle, J. , K. L. Nelson , N. Z. Counts , and M. Yudell . 2020. “Population‐Based Approaches to Mental Health: History, Strategies, and Evidence.” Annual Review of Public Health 41: 201–221. 10.1146/annurev-publhealth-040119-094247. PMC8896325 31905323

[fsn371613-bib-0096] Rahman, I. , S. K. Biswas , and P. A. Kirkham . 2006. “Regulation of Inflammation and Redox Signaling by Dietary Polyphenols.” Biochemical Pharmacology 72, no. 11: 1439–1452. 16920072 10.1016/j.bcp.2006.07.004

[fsn371613-bib-0097] Rajaram, S. , J. Jones , and G. J. Lee . 2019. “Plant‐Based Dietary Patterns, Plant Foods, and Age‐Related Cognitive Decline.” Advances in Nutrition 10: S422–S436. 31728502 10.1093/advances/nmz081 PMC6855948

[fsn371613-bib-0098] Ramis, M. , F. Sarubbo , D. Moranta , et al. 2020. “Cognitive and Neurochemical Changes Following Polyphenol‐Enriched Diet in Rats.” Nutrients 13: 59. In: s Note: MDPI stays neu‐tral with regard to jurisdictional claims in …. 33375450 10.3390/nu13010059 PMC7824548

[fsn371613-bib-0099] Ramis, M. R. , F. Sarubbo , S. Tejada , et al. 2020. “Chronic Polyphenon‐60 or Catechin Treatments Increase Brain Monoamines Syntheses and Hippocampal SIRT1 Levels Improving Cognition in Aged Rats.” Nutrients 12, no. 2: 326. 31991916 10.3390/nu12020326 PMC7071257

[fsn371613-bib-0100] Ransome, M. I. , and A. J. Hannan . 2013. “Impaired Basal and Running‐Induced Hippocampal Neurogenesis Coincides With Reduced Akt Signaling in Adult R6/1 HD Mice.” Molecular and Cellular Neuroscience 54: 93–107. 10.1016/j.mcn.2013.01.005. 23384443

[fsn371613-bib-0101] Rashet, A. , A. Abdi , and A. Barari . 2024. “Synergistic Role of Aerobic Training and Resveratrol on AMPK/PGC1‐α/SIRT1 Pathway in the Hippocampus of Rats With Alzheimer's Disease.” Journal of Archives in Military Medicine 12, no. 1: e144281.

[fsn371613-bib-0102] Ren, L. , Y. Zheng , L. Wu , et al. 2018. “Investigation of the Prevalence of Cognitive Impairment and Its Risk Factors Within the Elderly Population in Shanghai, China.” Scientific Reports 8, no. 1: 3575. 10.1038/s41598-018-21983-w. 29476112 PMC5824836

[fsn371613-bib-0103] Ribarič, S. 2022. “Physical Exercise, a Potential Non‐Pharmacological Intervention for Attenuating Neuroinflammation and Cognitive Decline in Alzheimer's Disease Patients.” International Journal of Molecular Sciences 23, no. 6: 3245. 10.3390/ijms23063245. 35328666 PMC8952567

[fsn371613-bib-0104] Ribeiro, F. F. , and S. Xapelli . 2021. “An Overview of Adult Neurogenesis.” Advances in Experimental Medicine and Biology 1331: 77–94. 10.1007/978-3-030-74046-7_7. 34453294

[fsn371613-bib-0105] Rossi, L. , S. Mazzitelli , M. Arciello , C. Capo , and G. Rotilio . 2008. “Benefits From Dietary Polyphenols for Brain Aging and Alzheimer's Disease.” Neurochemical Research 33: 2390–2400. 18415677 10.1007/s11064-008-9696-7

[fsn371613-bib-0106] Sadeghi, T. K. , M. Taheri , and K. Irandoost . 2022. “The Effect of Intermittent Exercise and Quercetin Supplementation on Cognitive Factors Affecting BDNF and CREB in the Brain Hippocampus of Rats With Colon Cancer.” Journal of Sport and Motor Development and Learning 14, no. 2: 34–53.

[fsn371613-bib-0107] Sadighparvar, S. , S. G. Darband , B. Yousefi , et al. 2020. “Combination of Quercetin and Exercise Training Attenuates Depression in Rats With 1,2‐Dimethylhydrazine‐Induced Colorectal Cancer: Possible Involvement of Inflammation and BDNF Signalling.” Experimental Physiology 105, no. 9: 1598–1609. 10.1113/ep088605. 32681548

[fsn371613-bib-0108] Salta, E. , O. Lazarov , C. P. Fitzsimons , R. Tanzi , P. J. Lucassen , and S. H. Choi . 2023. “Adult Hippocampal Neurogenesis in Alzheimer's Disease: A Roadmap to Clinical Relevance.” Cell Stem Cell 30, no. 2: 120–136. 10.1016/j.stem.2023.01.002. 36736288 PMC10082636

[fsn371613-bib-0109] Sanaeifar, F. , S. Pourranjbar , M. Pourranjbar , et al. 2024. “Beneficial Effects of Physical Exercise on Cognitive‐Behavioral Impairments and Brain‐Derived Neurotrophic Factor Alteration in the Limbic System Induced by Neurodegeneration.” Experimental Gerontology 195: 112539. 10.1016/j.exger.2024.112539. 39116955

[fsn371613-bib-0110] Sarubbo, F. , D. Moranta , and G. Pani . 2018. “Dietary Polyphenols and Neurogenesis: Molecular Interactions and Implication for Brain Ageing and Cognition.” Neuroscience and Biobehavioral Reviews 90: 456–470. 10.1016/j.neubiorev.2018.05.011. 29753753

[fsn371613-bib-0111] Seki, T. , T. Hori , H. Miyata , M. Maehara , and T. Namba . 2019. “Analysis of Proliferating Neuronal Progenitors and Immature Neurons in the Human Hippocampus Surgically Removed From Control and Epileptic Patients.” Scientific Reports 9, no. 1: 18194. 10.1038/s41598-019-54684-z. 31796832 PMC6890740

[fsn371613-bib-0112] Selles, M. C. , M. M. Oliveira , and S. T. Ferreira . 2018. “Brain Inflammation Connects Cognitive and Non‐Cognitive Symptoms in Alzheimer's Disease.” Journal of Alzheimer's Disease 64, no. s1: S313–s327. 10.3233/jad-179925. 29710716

[fsn371613-bib-0113] Shalan, N. A. , N. A. Rahim , and N. I. Mohamad . 2021. “Exercise and Supplementation of Black Mulberry Fruit Extract, Sunflower Seed and Pumpkin Seed Enhance Cognitive Performance Among Sedentary University Students.” Current Nutrition & Food Science 17, no. 1: 105–110.

[fsn371613-bib-0114] Shalan, N. A. A. M. , N. A. Rahim , and N. Saad . 2020. “The Effects of Black Mulberry Fruit Extract, Sunflower Seed, and Pumpkin Seed With Exercise on Memory Function and Neural Activation Biomarkers Among Healthy Young Adults.” Current Research in Nutrition and Food Science Journal 8, no. 1: 281–290.

[fsn371613-bib-0115] Silva, R. F. , and L. Pogačnik . 2020. “Polyphenols From Food and Natural Products: Neuroprotection and Safety.” Antioxidants 9, no. 1: 61. 31936711 10.3390/antiox9010061 PMC7022568

[fsn371613-bib-0116] Slotnick, S. D. 2022. “The Hippocampus and Long‐Term Memory.” Cognitive Neuroscience 13: 113–114. 10.1080/17588928.2022.2128736. 36165735

[fsn371613-bib-0117] Spalding, K. L. , O. Bergmann , K. Alkass , et al. 2013. “Dynamics of Hippocampal Neurogenesis in Adult Humans.” Cell 153, no. 6: 1219–1227. 10.1016/j.cell.2013.05.002. 23746839 PMC4394608

[fsn371613-bib-0118] Suganuma, M. , S. Okabe , M. Oniyama , Y. Tada , H. Ito , and H. Fujiki . 1998. “Wide Distribution of [3H](‐)‐Epigallocatechin Gallate, a Cancer Preventive Tea Polyphenol, in Mouse Tissue.” Carcinogenesis 19, no. 10: 1771–1776. 9806157 10.1093/carcin/19.10.1771

[fsn371613-bib-0119] Suijo, K. , S. Inoue , Y. Ohya , et al. 2013. “Resistance Exercise Enhances Cognitive Function in Mouse.” International Journal of Sports Medicine 34, no. 4: 368–375. 10.1055/s-0032-1323747. 23041964

[fsn371613-bib-0120] Sun, H. , J. Zhang , L. Zhang , H. Liu , H. Zhu , and Y. Yang . 2010. “Environmental Enrichment Influences BDNF and NR1 Levels in the Hippocampus and Restores Cognitive Impairment in Chronic Cerebral Hypoperfused Rats.” Current Neurovascular Research 7, no. 4: 268–280. 10.2174/156720210793180819. 20854252

[fsn371613-bib-0121] Sun, L. , K. Cui , F. Xing , and X. Liu . 2020. “Akt Dependent Adult Hippocampal Neurogenesis Regulates the Behavioral Improvement of Treadmill Running to Mice Model of Post‐Traumatic Stress Disorder.” Behavioural Brain Research 379: 112375. 10.1016/j.bbr.2019.112375. 31759046

[fsn371613-bib-0122] Sun, L. N. , J. S. Qi , and R. Gao . 2018. “Physical Exercise Reserved Amyloid‐Beta Induced Brain Dysfunctions by Regulating Hippocampal Neurogenesis and Inflammatory Response via MAPK Signaling.” Brain Research 1697: 1–9. 10.1016/j.brainres.2018.04.040. 29729254

[fsn371613-bib-0123] Tangsaengvit, N. , W. Kitphati , S. Tadtong , N. Bunyapraphatsara , and V. Nukoolkarn . 2013. “Neurite Outgrowth and Neuroprotective Effects of Quercetin From Caesalpinia Mimosoides Lamk. On Cultured P19‐Derived Neurons.” Evidence‐Based Complementary and Alternative Medicine 2013, no. 1: 838051. 23840266 10.1155/2013/838051 PMC3693115

[fsn371613-bib-0124] Tartt, A. N. , C. A. Fulmore , Y. Liu , et al. 2018. “Considerations for Assessing the Extent of Hippocampal Neurogenesis in the Adult and Aging Human Brain.” Cell Stem Cell 23, no. 6: 782–783. 10.1016/j.stem.2018.10.025. 30526880 PMC6830306

[fsn371613-bib-0125] Teixeira, A. M. , C. S. Pase , N. Boufleur , et al. 2011. “Exercise Affects Memory Acquisition, Anxiety‐Like Symptoms and Activity of Membrane‐Bound Enzyme in Brain of Rats Fed With Different Dietary Fats: Impairments of Trans Fat.” Neuroscience 195: 80–88. 10.1016/j.neuroscience.2011.08.055. 21893165

[fsn371613-bib-0126] Toda, T. , S. L. Parylak , S. B. Linker , and F. H. Gage . 2019. “The Role of Adult Hippocampal Neurogenesis in Brain Health and Disease.” Molecular Psychiatry 24, no. 1: 67–87. 10.1038/s41380-018-0036-2. 29679070 PMC6195869

[fsn371613-bib-0127] Toktam‐Barmar, Z. , S. Cheragh‐Birjandi , and N. Rezaeian . 2022. “High Intensity Aerobic Interval Training and Curcumin Supplementation Could Control Hippocampal Neurotoxicity Induced by Oxygenated Water Consumption in Male Rats.” Ilam‐University‐of‐Medical‐Sciences 9, no. 4: 12.

[fsn371613-bib-0128] Trevizol, L. , L. Bassôa , I. Fraga , et al. 2018. “Grape Juice Consumption and/or Exercise Training‐Induced Neuroplasticity and Memory Improvement in Healthy Elderly Women.” Journal of Cellular and Molecular Physiology 2: 154–163.

[fsn371613-bib-0129] Vafaee, R. , H. Soori , M. Hedayati , E. Ainy , and H. Hatamabadi . 2019. “Effects of Resveratrol Supplementation in Male Wistar Rats Undergoing an Endurance Exercise and Acute Exercise Training.” Human Antibodies 27, no. 4: 257–264. 10.3233/HAB-190380. 31127758

[fsn371613-bib-0130] Valipour Dehnou, V. 2024. “The Effect of Saffron Extract During Resistance Training on BDNF Protein Expression in the Hippocampus of Rats With Type 2 Diabetes.” Iranian Journal of Diabetes and Metabolism 24, no. 5: 302–309.

[fsn371613-bib-0131] Valls‐Pedret, C. , R. M. Lamuela‐Raventós , A. Medina‐Remon , et al. 2012. “Polyphenol‐Rich Foods in the Mediterranean Diet Are Associated With Better Cognitive Function in Elderly Subjects at High Cardiovascular Risk.” Journal of Alzheimer's Disease 29, no. 4: 773–782. 10.3233/JAD-2012-111799 22349682

[fsn371613-bib-0132] Van Praag, H. , B. R. Christie , T. J. Sejnowski , and F. H. Gage . 1999. “Running Enhances Neurogenesis, Learning, and Long‐Term Potentiation in Mice.” Proceedings of the National Academy of Sciences 96, no. 23: 13427–13431. 10.1073/pnas.96.23.13427 PMC23964 10557337

[fsn371613-bib-0133] Vignes, M. , T. Maurice , F. Lanté , et al. 2006. “Anxiolytic Properties of Green Tea Polyphenol (‐)‐Epigallocatechin Gallate (EGCG).” Brain Research 1110, no. 1: 102–115. 16859659 10.1016/j.brainres.2006.06.062

[fsn371613-bib-0134] Villalba, A. , M. Götz , and V. Borrell . 2021. “The Regulation of Cortical Neurogenesis.” Current Topics in Developmental Biology 142: 1–66. 10.1016/bs.ctdb.2020.10.003. 33706916

[fsn371613-bib-0135] Vivar, C. , M. C. Potter , and H. van Praag . 2013. “All About Running: Synaptic Plasticity, Growth Factors and Adult Hippocampal Neurogenesis.” Current Topics in Behavioral Neurosciences 15: 189–210. 10.1007/7854_2012_220. 22847651 PMC4565722

[fsn371613-bib-0136] Wahl, D. , V. C. Cogger , S. M. Solon‐Biet , et al. 2016. “Nutritional Strategies to Optimise Cognitive Function in the Aging Brain.” Ageing Research Reviews 31: 80–92. 27355990 10.1016/j.arr.2016.06.006 PMC5035589

[fsn371613-bib-0137] Wu, A. , and J. Zhang . 2023. “Neuroinflammation, Memory, and Depression: New Approaches to Hippocampal Neurogenesis.” Journal of Neuroinflammation 20, no. 1: 283. 10.1186/s12974-023-02964-x. 38012702 PMC10683283

[fsn371613-bib-0138] Yang, N. , X. Liu , X. Niu , et al. 2022. “Activation of Autophagy Ameliorates Age‐Related Neurogenesis Decline and Neurodysfunction in Adult Mice.” Stem Cell Reviews and Reports 18, no. 2: 626–641. 10.1007/s12015-021-10265-0. 34546510

[fsn371613-bib-0139] Youdim, K. A. , M. S. Dobbie , G. Kuhnle , A. R. Proteggente , N. J. Abbott , and C. Rice‐Evans . 2003. “Interaction Between Flavonoids and the Blood–Brain Barrier: In Vitro Studies.” Journal of Neurochemistry 85, no. 1: 180–192. 12641740 10.1046/j.1471-4159.2003.01652.x

[fsn371613-bib-0140] Zhang, F. , H. Wang , Q. Wu , et al. 2013. “Resveratrol Protects Cortical Neurons Against Microglia‐Mediated Neuroinflammation.” Phytotherapy Research 27, no. 3: 344–349. 22585561 10.1002/ptr.4734

[fsn371613-bib-0141] Zhang, Z. , H. Hamada , and P. M. Gerk . 2019. “Selectivity of Dietary Phenolics for Inhibition of Human Monoamine Oxidases A and B.” BioMed Research International 2019, no. 1: 8361858. 30809547 10.1155/2019/8361858 PMC6364133

[fsn371613-bib-0142] Zhao, X. , and H. van Praag . 2020. “Steps Towards Standardized Quantification of Adult Neurogenesis.” Nature Communications 11, no. 1: 4275. 10.1038/s41467-020-18046-y. PMC7450090 32848155

[fsn371613-bib-0143] Zhou, Y. , X. Wang , Y. Liu , et al. 2023. “Mechanisms of Abnormal Adult Hippocampal Neurogenesis in Alzheimer's Disease.” Frontiers in Neuroscience 17: 1125376. 10.3389/fnins.2023.1125376. 36875663 PMC9975352

